# The Role of Genistein in Type 2 Diabetes and Beyond: Mechanisms and Therapeutic Potential

**DOI:** 10.3390/molecules30051068

**Published:** 2025-02-26

**Authors:** Mateusz Kciuk, Weronika Kruczkowska, Katarzyna Wanke, Julia Gałęziewska, Damian Kołat, Somdutt Mujwar, Renata Kontek

**Affiliations:** 1 Department of Molecular Biotechnology and Genetics, Univeristy of Lodz, Banacha 12/16, 90-237 Lodz, Poland; katarzyna.wanke@edu.uni.lodz.pl (K.W.); renata.kontek@biol.uni.lodz.pl (R.K.); 2 Department of Functional Genomics, Faculty of Medicine, Medical University of Lodz, Żeligowskiego 7/9, 90-752 Lodz, Poland; weronika.kruczkowska@stud.umed.lodz.pl (W.K.); julia.galeziewska@stud.umed.lodz.pl (J.G.); damian.kolat@umed.lodz.pl (D.K.); 3 Department of Biomedicine and Experimental Surgery, Faculty of Medicine, Medical University of Lodz, Narutowicza 60, 90-136 Lodz, Poland; 4 Chitkara College of Pharmacy, Chitkara University, Rajpura 140401, Punjab, India; somduttmujwar@gmail.com

**Keywords:** anti-oxidant, bioavailability, genistein, metabolic disease, natural product, type 2 diabetes mellitus

## Abstract

The global prevalence of type 2 diabetes mellitus (T2DM) necessitates the exploration of novel therapeutic approaches to mitigate its complex molecular pathogenesis. This review investigates the potential role of genistein, a prominent isoflavone derived from soybeans, in the management of T2DM. Recognized for its selective estrogen receptor modulator (SERM) activity, genistein exerts a multifaceted influence on key intracellular signaling pathways, which are crucial in regulating cell proliferation, apoptosis, and insulin signaling. Genistein’s anti-inflammatory, anti-oxidant, and metabolic regulatory properties position it as a promising candidate for T2DM intervention. This review synthesizes current research spanning preclinical studies and clinical trials, emphasizing genistein’s impact on insulin sensitivity, glucose metabolism, and inflammatory markers. Additionally, this review addresses genistein’s bioavailability, safety, and potential influence on gut microbiota composition. By consolidating these findings, this review aims to provide a comprehensive understanding of genistein’s therapeutic potential in T2DM management, offering valuable insights for future research and clinical practice.

## 1. Introduction

Soybean (*Glycine max* (L.) Merr.) is globally valued for its exceptional oil and protein content, which underscores its significant economic and nutritional roles. Cultivated for thousands of years, with origins traced primarily to ancient China, soybean holds historical prominence as one of the “Five Sacred Grains” alongside barley, millet, wheat, and rice. As a highly versatile crop, soybean serves as a foundational ingredient in a range of food products, including soy milk and tofu, and its processing generates oil, bran, flour, soluble extracts, and textured vegetable protein (TVP). Soy and soy products are prevalent dietary options for vegetarians, owing to their substantial protein content and adaptability in creating meat alternatives and dairy substitutes. Despite these diverse applications, soybeans remain underutilized in both industrial contexts and global human diets, suggesting untapped potential for broader exploitation. Recently, the growing focus on plant-derived bioactives has driven an expansion of soy products in the functional food market, reflecting a paradigm shift toward dietary components enriched with phytochemicals and other bioactive compounds. Nonetheless, there are concerns over the potential health impacts, including the efficacy in reducing cardiovascular risk and, conversely, the possible disturbance of thyroid function and sexual hormones [1,2,3].

**Figure 1:**
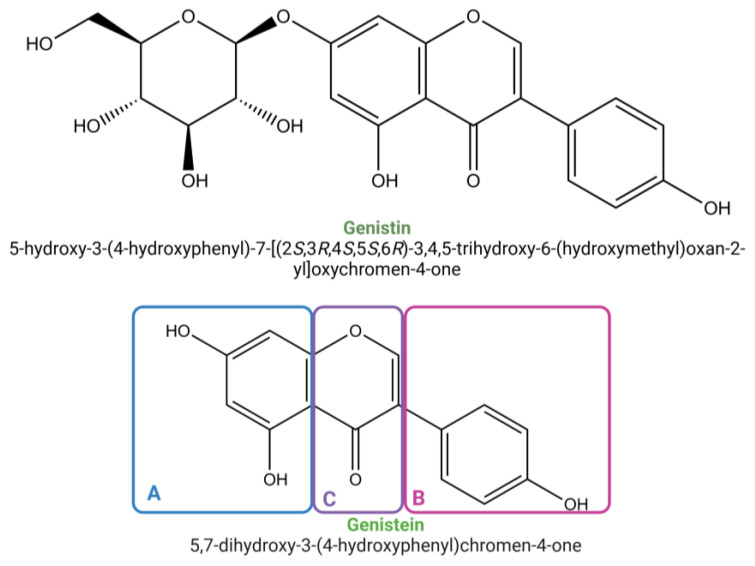
Chemical structures of genistin and genistein. Both of the compounds contain two aromatic benzene rings (A and B) and a non-aromatic heterocyclic pyran ring (C), forming a 3-phenylchromen-4-one backbone. Created in BioRender. Kciuk, M. (2025) https://BioRender.com/n34w609 (accessed on 17 February 2025).

The soy constituents that have garnered the most study interest are isoflavones, polyphenols with significant estrogenic effects found abundantly in soybeans. Genistein (5,7,4′-trihydroxy isoflavone) is a prominent example of this type of compound with well-documented health-promoting properties. Genistin, the predominant glycosylated form of genistein in natural sources, primarily exists in esterified forms such as malonyl-genistin (6′-O-malonylgenistin) and, to a lesser extent, acetyl-genistin (6′-O-acetylgenistin). These derivatives undergo enzymatic or heat-induced hydrolysis during food processing, leading to the release of genistin, which can further degrade into genistein—a compound characterized by its low molecular weight (270.24 Dalton) and a unique structure consisting of two aromatic benzene rings (A and B) and a non-aromatic heterocyclic pyran ring (C), forming a 3-phenylchromen-4-one backbone. The hydroxyl groups located at the 4′, 5, and 7 positions play a crucial role in its pronounced anti-oxidant activity (Figure 1) [4,5,6,7].

This conversion is particularly pronounced at elevated temperatures (e.g., 80 °C), where malonyl-genistin readily loses its malonyl group. Acylation plays a crucial role in modulating the bioavailability and metabolism of isoflavones, including genistin. By increasing lipophilicity, acylation can enhance gastrointestinal absorption while also improving stability against enzymatic and chemical degradation. Moreover, acylated isoflavones follow distinct metabolic pathways compared to their non-acylated counterparts, influencing interactions with key enzymes such as intestinal β-glucosidases and hepatic enzymes. These metabolic modifications can ultimately impact enterohepatic circulation and systemic bioavailability [4,5,6,7,8].

The content of genistein and its various forms (aglycone, glucoside, and esters) varies significantly across different soy-based food products. In soybeans, soy nuts, and soy powder, the levels of genistein and its β-glucoside conjugate, genistin, range from 4.6 to 18.2 μg/g and 200.6 to 968.1 μg/g, respectively. Soy milk and tofu contain lower amounts, with genistein levels of 1.9 to 13.9 μg/g and genistin levels of 94.8 to 137.7 μg/g. Fermented soybean products show higher concentrations of the aglycone form. In miso and natto, genistein levels range from 38.5 to 229.1 μg/g, while genistin levels are between 71.7 and 492.8 μg/g. This increase in genistein content is attributed to the cleavage of the β-glycosyl bond of genistin by microbes during fermentation. For a summary of the content of genistein in different food products, see the tables provided by other authors [9,10].

Recognized as a selective estrogen receptor modulator (SERM), genistein exhibits a preferential affinity for estrogen receptor (ER)β over ERα, triggering signaling pathways that regulate cell proliferation and apoptosis. This pleiotropic activity is mediated through its modulation of key intracellular pathways, including the nuclear factor kappa-light-chain-enhancer of activated B cells (NF-κB), phosphatidylinositol 3-kinase (PI3K)/protein kinase B (PKB/AKT), and its influence on gene and protein expression. Genistein’s anti-cancer, anti-oxidant, anti-inflammatory, anti-angiogenic, and pro-apoptotic effects further underline its potential as a versatile bioactive compound. Despite its biological activity, GEN is poorly soluble in water but demonstrates good solubility in polar solvents like acetone and ethanol [11,12].

Type 2 diabetes mellitus (T2DM) involves complex molecular disturbance centered around insulin resistance and pancreatic β-cell dysfunction. At the cellular level, insulin resistance develops when cells become less responsive to insulin signaling, primarily through the disruption of the PI3K/AKT pathway. This disruption reduces glucose transporter (GLUT) expression and glucose uptake in peripheral tissues [13,14,15]. Chronic inflammation, marked by elevated cytokines like interleukin 6 (IL-6), tumor necrosis factor-alpha (TNF-α), and C-reactive protein (CRP), further suppresses insulin signaling [16]. Pancreatic β-cells initially compensate for insulin resistance by increasing insulin production. However, this compensation eventually fails due to progressive β-cell dysfunction, characterized by cellular senescence, mitochondrial dysfunction, and oxidative stress damage. The deterioration of β-cells results in inadequate insulin secretion despite elevated blood glucose levels [14,17]. Adipose tissue plays a crucial role through multiple mechanisms. Dysfunctional adipocytes exhibit altered adipokine secretion, impaired adipogenesis, and enhanced inflammatory cytokine production. Adipocyte senescence contributes to chronic low-grade inflammation or “inflammaging,” creating a positive feedback loop that worsens insulin resistance [18,19]. Genetic factors significantly influence T2DM development through variations in key genes. Fat mass and obesity-associate genes (*FTO*) affect obesity risk and energy regulation, peroxisome proliferator-activated receptor gamma (*PPARG*) impacts glucose regulation and cellular differentiation, and insulin receptor substrate 1 (*IRS1*) modulates insulin signaling pathways. Other genes like transcription factor 7 like 2 (*TCF7L2*), ATP binding cassette subfamily C member 8 (*ABCC8*), and hepatocyte nuclear factor-1 alpha (*HNF1A*) influence pancreatic β-cell function and insulin secretion [20]. The disease progression involves metabolic disruption across multiple tissues. The liver increases glucose production despite hyperglycemia, while peripheral tissues show reduced glucose uptake. Mitochondrial dysfunction and oxidative stress further compromise cellular energy metabolism. The accumulation of senescent cells in metabolic tissues, characterized by the senescence-associated secretory phenotype (SASP), perpetuates inflammation and impairs tissue function and repair [21,22]. These interconnected molecular mechanisms create a complex pathophysiological environment that maintains chronic hyperglycemia and promotes the development of diabetic complications.

The increasing prevalence of T2DM globally and the complex molecular mechanisms involved in its pathogenesis create a need for exploring novel therapeutic approaches. Genistein has shown promising potential in mitigating various aspects of T2DM through its anti-inflammatory, anti-oxidant, and metabolic regulatory properties. Additionally, recent evidence suggests genistein may influence gut microbiota composition and insulin sensitivity. However, a comprehensive understanding of its bioavailability, safety, and clinical efficacy is crucial for its potential therapeutic application. This review aims to consolidate and analyze existing research on genistein’s role in T2DM management, from preclinical studies to clinical trials, while also addressing important aspects of its bioavailability and safety. This structured approach helps bridge the gap between laboratory findings and clinical applications, providing valuable insights for both researchers and healthcare practitioners considering genistein as a therapeutic option for T2DM.

## 2. Preclinical Studies

Genistein has shown promise in managing T2DM. Its beneficial effects stem from a multifaceted approach that targets several key aspects of the disease. Firstly, genistein supports the health and function of pancreatic beta cells. It encourages the growth of these cells while protecting them from damage and death. This is crucial as beta cell dysfunction is a hallmark of T2DM. Moreover, genistein enhances the ability of beta cells to release insulin in response to rising blood sugar levels, a critical step in maintaining glucose control [23,24]. Beyond its direct impact on beta cells, genistein exerts a profound influence on metabolic pathways. It interacts with the cyclic adenosine monophosphate/protein kinase A (cAMP/PKA) signaling pathway, a crucial regulator of various metabolic processes, including insulin secretion and glucose homeostasis [25]. Interestingly, genistein can also modify the composition of gut microbiota, the complex community of microorganisms residing in the intestines. This interaction with the gut microbiome may further contribute to its beneficial effects on metabolism [26]. Inflammation plays a significant role in the development and progression of T2DM. Genistein exhibits potent anti-inflammatory properties, helping to reduce systemic inflammation and mitigate insulin resistance, a key feature of the disease [27].

In addition to its effects on glucose metabolism, genistein positively influences lipid metabolism. It promotes the breakdown of fatty acids for energy while simultaneously inhibiting the production of new fat and reducing the release of pro-inflammatory molecules from fat tissue. This dual action helps improve blood lipid profiles and reduces the risk of fatty liver, a common complication of diabetes [28,29]. Recent research suggests that genistein may also exert its effects through the central nervous system. It can influence the activity of brain regions involved in metabolic control, potentially impacting energy balance and insulin sensitivity [30,31,32].

## 3. Clinical Studies

Inflammation serves as a pivotal link between diabetes and end-stage renal disease (ESRD), contributing to the progression and complications of both conditions. Chronic low-grade inflammation is a hallmark of T2DM, driven by hyperglycemia-induced oxidative stress and the accumulation of advanced glycation end-products (AGEs). These processes activate pro-inflammatory pathways, resulting in the release of cytokines such as TNF-α and IL-6 [13,33]. In diabetic nephropathy, prolonged hyperglycemia damages glomerular structures, leading to proteinuria, fibrosis, and eventual renal failure [34]. As diabetes remains the leading cause of ESRD globally, the systemic inflammation associated with both conditions is exacerbated by the accumulation of uremic toxins in ESRD patients, further amplifying pro-inflammatory cytokine production and oxidative stress. Moreover, shared mechanisms, such as endothelial dysfunction and immune dysregulation, contribute to vascular damage and heightened cardiovascular risk in this population. Elevated inflammatory markers, including CRP and IL-6, are strongly associated with poor outcomes in patients with diabetes and ESRD [35,36].

Fanti et al. investigated the potential anti-inflammatory and nutritional benefits of dietary soy isoflavones in hemodialysis (HD) patients with elevated systemic inflammation, as indicated by high CRP levels. This study found that supplementation with soy-based isoflavone-rich nutritional products significantly increased serum isoflavone levels in the soy group compared to the control group, with a 5- to 10-fold elevation (e.g., median genistein: 337.9 nM in the soy group vs. 41.4 nM in the control group). Notably, a strong inverse correlation was observed between changes in individual serum isoflavone levels (Δ-isoflavone) and CRP levels in the soy group, suggesting potential anti-inflammatory effects. Furthermore, Δ-isoflavone was positively correlated with changes in nutritional markers, such as albumin and insulin-like growth factor-1 (IGF-1). Although group-level CRP reductions were not statistically significant, a trend towards lower CRP levels was noted in the soy group after 8 weeks, in contrast to the control group. These findings suggest that soy isoflavones may offer promising benefits in modulating inflammation and improving nutritional status in HD patients with systemic inflammation, warranting further investigation in larger studies. The findings of this study have significant implications for diabetic patients, particularly those on HD who often exhibit systemic inflammation and malnutrition as part of their disease profile. Since diabetes is the leading cause of ESRD, dietary interventions with isoflavone-rich soy may serve as an adjunctive strategy to reduce inflammation, improve nutritional status, and potentially mitigate complications in this high-risk population. Further research in the diabetic patients population specifically, is warranted to confirm these benefits and explore the broader applicability of soy-based interventions in their clinical management [37].

Another study evaluated the effects of high-dose isoflavones on inflammatory and metabolic markers in 75 healthy, normal-weight postmenopausal women. Participants were randomized to receive either a soy protein supplement containing 160 mg of isoflavones or a soy protein placebo for 12 weeks. The results revealed a significant increase in serum adiponectin levels within the isoflavone group compared to the placebo group. Adiponectin, an anti-inflammatory adipokine, is known to enhance insulin sensitivity and regulate glucose metabolism, suggesting potential metabolic benefits. However, no significant changes were observed in other parameters, such as glucose, insulin, or other inflammatory cytokines, between the groups. Overall, these findings suggest that while isoflavones may influence adiponectin levels, their impact on broader metabolic and inflammatory markers in healthy, normal-weight postmenopausal women is limited. Isoflavones may help mitigate the inflammatory and metabolic disruptions associated with menopause and T2DM, warranting further exploration in diabetic populations to determine their efficacy in improving glycemic control and reducing diabetes-related complications [38].

In the preliminary study, Nebbioso et al. investigated the effects of systemic oral anti-oxidant (AO) treatment on oxidative stress and retinal function in patients with preretinopathic diabetes (PRD). Thirty-two PRD patients with good metabolic control were randomized to receive either AO treatment, consisting of α-lipoic acid (400 mg/day) combined with genistein and vitamins, or a placebo for 30 days. Plasma oxidative stress levels were assessed using the Free Radical Analytical System, and retinal function was evaluated through full-field electroretinogram (ERG) measurements. This study found a statistically significant increase in plasma AO levels and improved ERG oscillatory potential values in the AO treatment group, whereas no such changes were observed in the placebo group. These results suggest that oral AO supplementation may protect retinal cells in PRD patients by enhancing the plasma AO barrier and improving retinal electrophysiological responses. These findings have significant implications for diabetic patients, particularly those at risk or in the early stages of diabetic retinopathy. Oxidative stress is a key factor in the development and progression of diabetic retinopathy, as it contributes to retinal damage and vascular dysfunction. The observed increase in plasma anti-oxidant levels and improvement in ERG oscillatory potentials indicate that systemic AO treatment may offer retinal protection by counteracting oxidative stress and preserving retinal function [39].

A double-blind, placebo-controlled trial evaluated the effects of genistein on metabolic and cardiovascular risk factors in 120 postmenopausal women with metabolic syndrome (MetS). Participants were assigned to receive either 54 mg of genistein daily or a placebo for one year. This study demonstrated significant improvements in several metabolic and cardiovascular parameters in the genistein group. Fasting glucose, fasting insulin, and insulin resistance (HOMA-IR; homeostatic model assessment of insulin resistance) decreased markedly in genistein recipients while remaining unchanged in the placebo group. Additionally, genistein significantly increased high-density lipoprotein cholesterol (HDL-C; mean from 46.4 to 56.8 mg/dL) and adiponectin levels, while reducing total cholesterol, low-density lipoprotein cholesterol (LDL-C; mean from 108.8 to 78.7 mg/dL), triglycerides, visfatin, and homocysteine (mean from 14.3 to 11.7 μmol/L). Blood pressure (both systolic and diastolic) was also reduced. Importantly, genistein was well tolerated, with no significant difference in adverse events between the genistein and placebo groups. These findings suggest that genistein could serve as an effective intervention for improving metabolic and cardiovascular health in postmenopausal women with MetS, a condition that predisposes individuals to T2DM and cardiovascular disease. The reduction in insulin resistance (HOMA-IR) and fasting glucose, coupled with favorable changes in lipid profiles and adipokines, such as adiponectin, indicates that genistein may enhance glycemic control and mitigate cardiovascular risk. Given the high prevalence of MetS among postmenopausal women and its strong association with diabetes progression, genistein presents a promising adjunctive therapy for diabetic patients, especially those struggling with comorbid metabolic and cardiovascular disorders. Future studies in diabetic populations are warranted to confirm these benefits and explore potential mechanisms of action [40].

A randomized, double-blind, placebo-controlled clinical trial evaluated the effects of genistein supplementation on metabolic parameters, oxidative stress, and obesity indices in 54 postmenopausal women with T2DM. Participants were randomly assigned to receive either two genistein capsules daily (54 mg per capsule; *n* = 28) or placebo capsules (*n* = 26) for 12 weeks. Genistein supplementation significantly reduced fasting blood glucose (FBS), glycated hemoglobin (A1C), serum triglycerides (TG), and malondialdehyde (MDA), a marker of oxidative stress, while increasing the total anti-oxidant capacity (TAC) compared to the placebo group. Additionally, HDL-C and insulin sensitivity, measured by the quantitative insulin sensitivity check index (QUICKI), significantly improved within the genistein group. However, changes in anthropometric indices and other variables were not significant in either group. These results indicate that genistein supplementation positively impacts glycemic control, lipid profiles, and oxidative stress markers in postmenopausal women with T2DM [41].

A randomized dietary intervention trial assessed the effects of a legume-based commercial food product on anthropometric and biochemical parameters in 60 healthy university students (53% male). Participants in the study group consumed 15 g of the product daily for three months, administered in cycles of five days on and two days off. The intervention resulted in significant reductions in serum glucose levels, MDA, and the HOMA-IR. However, no significant changes were observed in other parameters, including anthropometric measures or lipid profiles. The improvements in glucose metabolism, oxidative stress, and insulin resistance are likely attributable to the bioactive compounds in legumes, including polyphenols and isoflavones such as genistein [42].

Another study investigated the effects of genistein on insulin resistance, gut microbiota composition, and metabolic parameters in 45 participants with obesity (body mass index (BMI) ≥ 30 and ≤40 kg/m^2^) and insulin resistance (HOMA-IR > 2.5). Participants were randomly assigned to receive either a placebo or genistein capsules (50 mg/day) for 2 months. The results demonstrated that genistein supplementation significantly reduced insulin resistance, as measured by HOMA-IR and oral glucose tolerance tests. This improvement was accompanied by a notable modification in gut microbiota, with an increase in *Verrucomicrobia*, a phylum associated with improved metabolic health. Additionally, metabolic endotoxemia was reduced, and skeletal muscle gene expression related to fatty acid oxidation increased, as evidenced by enhanced 5′AMP-activated protein kinase (AMPK) phosphorylation and increased circulating metabolites of β-oxidation, acyl-carnitines, and ketone bodies. The findings suggest that genistein supplementation may offer a novel therapeutic strategy for improving insulin sensitivity in individuals with obesity, a key risk factor for T2DM. The observed changes in gut microbiota, particularly the increase in *Verrucomicrobia*, along with reduced metabolic endotoxemia and enhanced fatty acid oxidation in skeletal muscle, highlight the potential of genistein to address the metabolic dysfunctions that underlie insulin resistance and obesity. These results point to genistein as a promising dietary intervention for managing obesity-related metabolic abnormalities, potentially reducing the risk of developing T2DM. However, further long-term studies are needed to confirm these effects and establish the clinical benefits of genistein supplementation in diabetic populations [43].

A case–control study within the PREDIMED trial analyzed urinary phenolic compounds to identify metabolites associated with T2DM and fasting plasma glucose levels. A panel of 41 phenolic compounds was analyzed using a novel liquid chromatography–mass spectrometry method, and two metabolites—dihydrocaffeic acid and genistein diglucuronide—were identified as discriminants for T2DM. Both metabolites were associated with a lower risk of T2DM, with dihydrocaffeic acid additionally showing an inverse relationship with plasma glucose levels. Specifically, higher levels of dihydrocaffeic acid were linked to lower plasma glucose, while genistein diglucuronide was associated with reduced T2DM risk but did not directly affect plasma glucose. The findings suggest that specific phenolic metabolites, including genistein diglucuronide, could serve as biomarkers for T2DM risk and glucose regulation [44]. Table 1 provides a summary of clinical trials on genistein use in T2DM treatment.

**Figure 2:**
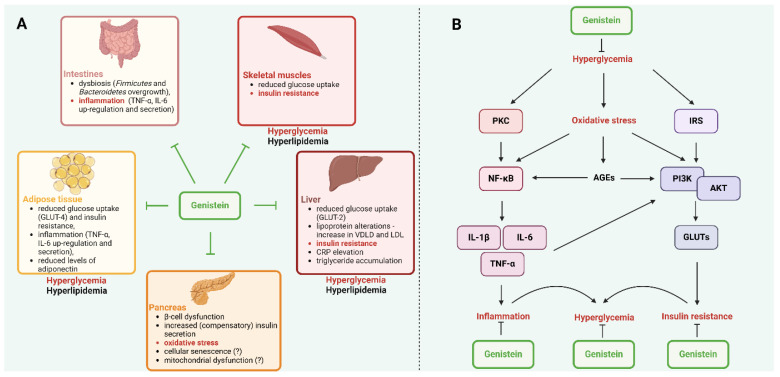
Pathological alterations associated with type II diabetes mellitus (T2DM) and the influence of genistein on the systemic basis (**A**) and molecular level (**B**) for the treatment of the disease. (**A**) In T2DM, there is a complex interplay between the pancreas, liver, skeletal muscles, adipose tissue, and intestinal microbiota, all contributing to the disease’s pathophysiology. The pancreas initially compensates for insulin resistance in peripheral tissues by increasing insulin secretion, but over time, beta-cell dysfunction leads to impaired insulin secretion. The liver exacerbates hyperglycemia by increasing gluconeogenesis due to insulin resistance, contributing to elevated fasting glucose levels. In skeletal muscles, reduced glucose uptake further worsens hyperglycemia, as muscles are major sites of insulin-mediated glucose disposal. Adipose tissue dysfunction in T2DM leads to altered secretion of adipokines like reduced adiponectin and increased inflammatory cytokines, which promote systemic insulin resistance. Additionally, altered intestinal microbiota composition, or dysbiosis, can influence inflammation and metabolic pathways, further impairing glucose metabolism and contributing to insulin resistance. This interconnected dysfunction among these organs and systems drives the progression and complications of T2DM. (**B**) In T2DM, chronic hyperglycemia triggers a cascade of metabolic disturbances, including oxidative stress and the formation of advanced glycation end products (AGEs). Oxidative stress, resulting from an imbalance between reactive oxygen species (ROS) and anti-oxidants, activates key signaling pathways such as protein kinase C (PKC) and nuclear factor kappa B (NF-κB). These pathways promote inflammation by upregulating pro-inflammatory cytokines like interleukins (ILs) and tumor necrosis factor-alpha (TNF-α). This inflammatory environment exacerbates insulin resistance by interfering with insulin signaling. Specifically, inflammation impairs the insulin receptor substrate (IRS) and its downstream signaling through phosphoinositide 3-kinase (PI3K) and AKT pathways, which are critical for glucose uptake. The disruption of this signaling cascade leads to reduced translocation of glucose transporter proteins (GLUTs), particularly GLUT4, to the cell membrane, diminishing glucose uptake in muscle and adipose tissues and perpetuating hyperglycemia and insulin resistance. Genistein was shown to impact particularly every aspect of these pathological changes, ameliorating hyperglycemia, hyperlipidemia, oxidative stress, inflammatory cascades, alleviating insulin resistance, or even contributing to the changes in microbiota composition. Created in BioRender. Kciuk, M. (2025) https://BioRender.com/v14w660 (accessed on 17 January 2025).

In summary, genistein’s beneficial effects on T2DM arise from a complex interplay of actions. However, further research is necessary to fully understand its mechanisms of action and translate these findings into effective clinical applications. Figure 2A indicates the pathological changes associated the T2DM and how genistin may exert the effect based on the systemic basis and (B) molecular level.

## 4. Bioavailability and Safety of Genistein

### 4.1. Bioavailability

While genistein exhibits a wide range of biological activities, including anti-cancer, anti-oxidant, anti-inflammatory effects, and potential anti-diabetic effects, its therapeutic efficacy is inherently linked to its bioavailability. Bioavailability encompasses the absorption, distribution, metabolism, and excretion of bioactive compounds, ultimately determining the concentration of genistein that reaches target tissues in its active form. Genistein’s bioavailability is influenced by factors such as its chemical modifications, intestinal absorption, conjugation into glucuronides and sulfates, and subsequent metabolic conversion by gut microbiota. Bioavailability studies are not merely supportive but integral to translating genistein’s biological activity into practical therapeutic applications. They inform the development of medical foods and nutraceuticals, ensuring consistent and effective dosing regimens for various human conditions. These findings will be thoroughly examined in the following chapter.

In the first human clinical study, genistein pharmacokinetics were characterized by rapid increases in urinary levels following soybean consumption, indicating efficient absorption and metabolism. Upon the administration of soy-containing genistein, urinary concentrations peaked within 16–24 h, followed by a gradual decline to slightly elevated baseline levels. This suggested rapid metabolic processing and the excretion of genistein and its derivatives. The urinary recovery of genistein, along with related isoflavonoids, was calculated at approximately 24.8% during an 88 h monitoring period. Free genistein aglycones were minimally present in urine, comprising only 0.1–2% of total urinary genistein, reflecting extensive conjugation and biotransformation. The observed kinetics correlated strongly with the administered soybean doses, further highlighting genistein’s predictable dose-dependent metabolic profile [45].

In the randomized crossover trial, the urinary excretion of genistein was significantly influenced by the type of soy product consumed. When participants consumed a tempeh-based diet (fermented soy), genistein recovery in urine was notably higher compared to the unfermented soybean pieces diet. This suggests that fermentation enhanced the bioavailability or absorption of genistein. Despite these differences, overall urinary isoflavonoid excretion (including genistein) was elevated in soy-supplemented diets compared to habitual diets [46].

Genistein excretion in urine was significantly influenced by diet composition, with higher levels observed in the legume/allium diet compared to the basal and high vegetable/fruit diets. The inclusion of legumes such as garbanzo beans in the diet appeared to promote greater isoflavonoid availability, including genistein. This pattern underscored the pivotal role of dietary legumes in enhancing isoflavonoid excretion. The variability in equol excretion, observed to be higher on the legume/allium and basal diets compared to the high vegetable/fruit diet, suggested that certain dietary components, such as legumes and milk-based products, may synergistically influence the metabolism of genistein and related isoflavonoids. These findings highlighted the impact of specific dietary patterns on the pharmacokinetics of genistein and its metabolic derivatives under controlled conditions [47].

Another study investigated the role of gut microbiota on the bioavailability of isoflavonoids, including genistein, in women following the consumption of soy milk containing isoflavones at varying doses (3.4, 6.9, or 10.3 µmol/kg body weight). Urinary and plasma measurements revealed significant interindividual variability in genistein metabolism, influenced by gut microflora activity. Among subjects excreting small amounts of fecal isoflavones, the urinary recovery of genistein averaged 10 ± 4% over 48 h, while those with high fecal isoflavone excretion showed significantly greater urinary recovery (37 ± 6%) and 2.5-fold higher plasma genistein concentrations at 24 h post-dose. These findings suggested that reduced intestinal degradation of genistein by gut microflora enhanced systemic bioavailability. Additionally, in vitro anaerobic incubation demonstrated a short intestinal half-life for genistein (3.3 h), highlighting rapid microbial metabolism as a key determinant of its bioavailability [48].

It was found that genistein is effectively absorbed from the gastrointestinal tract, assimilated by the liver, and eliminated in the bile as its 7-O-beta-glucuronide conjugate. Re-infused genistein 7-O-beta-glucuronide was effectively absorbed from the gastrointestinal tract, namely in the distal small intestine. In human individuals administered a soy beverage for two weeks, plasma concentrations of genistein and daidzein, measured by high-performance liquid chromatography (HPLC)–mass spectrometry (MS), varied from 0.55 to 0.86 µmol, predominantly as glucuronide and sulfate conjugates. This implies that genistein is efficiently absorbed from the small intestine and experiences enterohepatic circulation [49].

Tew et al. examined the impact of dietary fiber on the bioavailability of genistein in seven women following a single dose of isoflavones from tofu or TVP. A wheat fiber-supplemented diet containing 40 g of dietary fiber significantly reduced plasma genistein concentrations by 55% at 24 h post-dose and total urinary genistein excretion by 20% compared to a control diet with 15 g of dietary fiber. These findings indicate that the bulking and hydrophobic binding properties of insoluble wheat fiber may impede genistein absorption. Furthermore, urinary genistein excretion was observed to be 23% higher following tofu consumption compared to TVP, likely reflecting differences in the genistein content of the two soy products rather than variations in their chemical form. This highlights the influence of dietary components, such as fiber and soy food type, on the pharmacokinetics and bioavailability of genistein [50].

In contrast, another study aimed to characterize the bioavailability of soybean isoflavones, including genistein, as potential anti-carcinogenic food components. Eight women (aged 20–41 years) consumed varying doses of isoflavones (0.8–1.4 mg/kg body weight) from different soy foods such as soy milk, tofu, tempeh, cooked soybeans, or texturized vegetable protein. The urinary excretion of daidzein and genistein was measured over 24–48 h. After the consumption of background diets, the urinary recovery of daidzein was 26–27%, while genistein recovery was 18–20%. Notably, the urinary recovery of daidzein was consistently higher than that of genistein, ranging from 38 to 51% for daidzein and 9 to 16% for genistein from various soy foods. Plasma concentrations of both isoflavones were similar across different diet conditions, suggesting no significant effect of the background diet or food source on their bioavailability. The fecal recovery of isoflavones was minimal, likely due to bacterial metabolism. These findings indicated that isoflavone bioavailability is relatively stable, irrespective of the soy food source or dietary context, with daidzein showing higher urinary recovery compared to genistein [51].

A 1997 study evaluated the urinary excretion of genistein and other isoflavonoids in response to daily soy consumption containing 0–36 mg isoflavones, focusing on dose-dependency and differences between equol excreters and nonexcreters. The randomized, double-blind, crossover design included 14 participants, evenly split between equol excreters and nonexcreters. A highly linear dose–response relationship was observed for urinary genistein excretion relative to soy protein intake, with no significant differences between equol excreters and nonexcreters. These findings indicated that genistein bioavailability is primarily dose-dependent at low to moderate levels of soy consumption, irrespective of equol production status. Lignan excretion was unaffected by soy intake, highlighting the specificity of isoflavonoid responses to dietary soy. This study underscored the consistent pharmacokinetic behavior of genistein across varying levels of soy intake in diverse metabolic phenotypes [52].

Another pharmacokinetic study investigated genistein absorption, metabolism, and excretion in seven healthy male volunteers following ingestion of 60 g of *kinako* (baked soybean powder, containing 112 µmol genistein). Plasma genistein concentrations increased within 2 h, reaching a peak of 2.44 ± 0.65 µmol/L at 6 h and demonstrating a half-life of 8.36 h. Urinary excretion of genistein closely followed plasma trends, peaking at 1.1 µmol/h at 6 h post-ingestion. Fecal recovery was the primary route of excretion, with only 20.1% of ingested genistein recovered in urine and feces combined, indicating substantial metabolism or retention. High variability in plasma and urinary concentrations of genistein metabolites, such as equol and O-desmethylangolensin (O-DMA), reflected differences in metabolic phenotypes among subjects. The sustained plasma genistein levels suggest prolonged interaction with biological targets, supporting its potential pharmacologic effects after soy consumption [53].

It was revealed that, following ingestion, genistein is absorbed in the gastrointestinal tract and rapidly conjugated, with glucuronides (7- and 4′-glucuronides) predominating in circulation, while sulfates and the aglycone forms are present at much lower levels. Glucuronidation is catalyzed by multiple UDP-glucuronosyltransferase (UGT) isoforms, including UGT 1A1, 1A4, 1A6, 1A7, 1A9, and 1A10, with UGT 1A10—expressed in the human colon—being specific for genistein and indicating a significant role for intestinal glucuronidation during absorption. Post-absorption, hepatic, and renal microsomal UGTs also contributed to genistein glucuronidation, further metabolizing the absorbed aglycone. In contrast, sulfation is mediated by sulfotransferase (SULT) isoforms such as SULT 1A1*2, 1E, and 2A1, though this pathway is considerably less active, and several SULT isoforms exhibit no activity with genistein. These findings highlight the predominance of glucuronidation in genistein metabolism, particularly within the intestine during the first-pass phase, and underscore its systemic processing by hepatic and renal pathways. The conjugated metabolites, primarily glucuronides, ensure genistein’s efficient transport and excretion from the body [54].

While the above-mentioned studies focused on the pharmacokinetic parameters of genistein following the consumption of soy products, several studies aimed to assess the pharmacokinetics of purified compounds in human organisms. A study by Vänttinen et al. examined the transdermal absorption of genistein and daidzein applied to the skin in olive oil, with concentrations monitored in plasma and urine. Genistein exhibited a 3-fold higher plasma concentration compared to daidzein, whereas daidzein showed 2–3 times greater urinary excretion than genistein. Urinary recovery rates following the first application were 15.9% for daidzein and 7.7% for genistein, but these values significantly declined after repeated applications to 1.6% and 0.7%, respectively. This marked reduction in plasma and urinary concentrations suggested that both phytoestrogens, particularly genistein, may be retained in the skin with repeated transdermal exposure. These findings highlighted the potential for differential pharmacokinetics between plasma and urinary pathways and suggest skin storage as a key factor influencing isoflavone bioavailability in transdermal applications [55].

Busby et al. evaluated the effect of purified, unconjugated genistein and other isoflavones administered to healthy men at doses significantly higher than typical dietary intake. Two formulations, containing varying proportions of genistein, daidzein, and glycitein, were used to deliver genistein doses ranging from 1 to 16 mg/kg body weight. No clinically significant behavioral or physical changes were observed, though minor elevations in lipoprotein lipase and hypophosphatemia were noted without associated toxicity. Pharmacokinetic analysis revealed that free genistein had a mean elimination half-life of 3.2 h and a pseudo-half-life (total genistein) of 9.2 h. Isoflavones were rapidly cleared from plasma and excreted in urine, primarily as conjugates. These findings demonstrated that single high doses of purified genistein are well tolerated in humans and exhibit rapid absorption, metabolism, and excretion, supporting its safety in dietary supplement use at elevated doses [56].

Important insights regarding the metabolisms of genistein were proposed by Setchell et al. The authors found that isoflavone glycosides are not directly absorbed into the bloodstream. Instead, their bioavailability depends on the hydrolysis of the sugar moiety by intestinal beta-glucosidases, which facilitates uptake and the subsequent appearance of isoflavones in the peripheral circulation. This underscored the role of intestinal metabolism in the bioavailability of soy isoflavones [57].

Another study examined whether the hydrolysis of isoflavone glycosides to aglycones by β-glycosidase affects the pharmacokinetics of isoflavones in plasma and urine in postmenopausal women. Six postmenopausal European women participated in a randomized, double-blinded, cross-over trial where they consumed two soy-based drinks: one containing glycosides and the other hydrolyzed enzymatically to aglycones. Each drink provided 1 mg of total isoflavones per kg body weight and was consumed at one-week intervals. Plasma and urinary pharmacokinetics of daidzein, genistein, and glycitein were similar for both drinks, with plasma total isoflavone concentrations peaking at 4–5 µmol/L. Isoflavone secondary metabolites, including dihydrodaidzein, O-desmethylangolensin, equol, and dihydrogenistein, were identified in plasma and urine, but their relative concentrations varied across matrices. The findings revealed that enzymatic hydrolysis of glycosides to aglycones before consumption does not improve isoflavone bioavailability, demonstrating comparable absorption and retention profiles irrespective of the isoflavone form ingested [58].

Conflicting results have been reported on the bioavailability of isoflavones, such as genistein, in aglycone or glucoside forms across different populations. One study investigated genistein bioavailability in American women following ingestion of either form, accounting for typical dietary habits. Fifteen women (aged 46 ± 6 years) participated in a randomized, double-blind trial where blood samples were collected at multiple intervals over 48 h after consumption of isoflavone tablets with breakfast. Plasma curves for genistein were analyzed to determine the postprandial maximum concentration (C_max_), time to maximum concentration (t_max_), and area under the curve (AUC). Genistein concentrations exhibited two plasma peaks: an early peak at 1–2 h and a secondary peak at 4–8 h, indicative of enterohepatic circulation. Comparisons of C_max_, t_max_, and AUC values revealed no significant differences between the aglycone and glucoside forms, suggesting similar bioavailability for genistein regardless of form. These findings imply that, for genistein, the metabolic differences between the aglycone and glucoside forms observed in other isoflavones, such as daidzein, do not translate into significant variations in absorption or plasma exposure in American women. This supports the interchangeable use of genistein in either form for dietary or therapeutic purposes [59].

Another study examined the pharmacokinetics, safety, and in vivo biological activity of two preparations of unconjugated soy isoflavones, PTI G-2535 (43% genistein) and PTI G-4660 (90% genistein), in prostate cancer patients. Thirteen participants received single oral doses of each preparation (2, 4, or 8 mg/kg) with a one-week washout period between doses. The study revealed dose-dependent pharmacokinetic profiles, with C_max_ for total genistein ranging from 4.3 to 16.3 µmol and free genistein ranging from 0.066 to 0.17 µmol. PTI G-2535 exhibited a shorter half-life (15.03 h) and lower volume of distribution (189.9 L) compared to PTI G-4660 (22.41 h and 653.8 L, respectively). AUC values showed a trend toward higher exposure with PTI G-2535 at the 8 mg/kg dose. Treatment was well tolerated, with a single case of rash as the only reported toxicity [60].

Faughnan et al. conducted a study to investigate how urinary genistein excretion varies following the consumption of different soy-based foods and to examine the potential modifying effects of age and gender. The study included 40 participants: 20 men, 20 premenopausal women, and 17 postmenopausal women. Each participant consumed a single oral dose (0.44 mg isoflavones/kg body weight) of soy milk, TVP, or tempeh on three separate occasions. Complete 24 h urine samples were collected for four consecutive days during each testing period to evaluate genistein recovery. The results indicated that urinary genistein excretion was influenced by both gender and the food matrix. Women exhibited greater genistein recovery from soy milk compared to TVP, and premenopausal women had higher recovery from tempeh compared to soy milk. In contrast, no significant differences in genistein excretion across the soy foods were observed in men. Urinary daidzein excretion remained consistent across food types and was not significantly affected by age or gender. However, the conversion of daidzein to equol showed variation based on the food matrix, with equol producers exhibiting significantly higher excretion levels after consuming tempeh. These findings suggest that the fractional absorption of genistein is influenced by gender, food matrix, and the chemical composition of soy products [61].

A randomized, double-blind, placebo-controlled study evaluated the concentrations of genistein, daidzein, and equol in breast tissue homogenate, serum, and urine after supplementation with a soy-based food supplement. Nine women ingested the supplement, and nineteen received a placebo for five consecutive evenings before undergoing elective breast surgery. Tissue heterogeneity was accounted for by measuring markers of cellularity, epithelial content, vascular content, and fat composition. Urinary concentrations of genistein, daidzein, and equol were significantly elevated in the soy-supplemented group compared to placebo. In serum, only genistein concentrations were significantly higher in the soy group, while no significant differences in genistein, daidzein, or equol concentrations were observed in breast tissue homogenates between the two groups. Breast tissue concentrations of these analytes were in the low nanomolar range, markedly lower than serum concentrations, which were approximately 100 times higher. These findings suggest that short-term intake of soy-based supplements does not significantly increase phytoestrogen concentrations in breast tissue, even though systemic absorption is evident. The low tissue concentrations relative to serum raise questions about the biological relevance of soy-derived phytoestrogens in estrogen-sensitive tissues like the breast. Further studies are warranted to explore long-term supplementation and its potential effects on tissue distribution and estrogen receptor activity [62].

One study aimed to evaluate the health effects of isoflavone consumption on normal breast tissue by examining isoflavone concentrations, metabolites, biodistribution, and comparing them with 17β-estradiol exposure. Healthy women were randomly assigned to receive soy milk (*n* = 11), soy supplements (*n* = 10), or a control (*n* = 10). After a 4-day run-in period, participants consumed 3 daily doses of soy milk or supplements for 5 days before undergoing breast reduction surgery. Blood and breast tissue biopsies were analyzed using liquid chromatography–tandem mass spectrometry. Genistein and daidzein concentrations in serum ranged from 135.1 to 2831 nmol/L and 105.1 to 1397 nmol/L, respectively, and in breast tissue from 92.33 to 493.8 pmol/g and 22.15 to 770.8 pmol/g. The major metabolites were genistein-7-O-glucuronide and daidzein-7-O-glucuronide, with 98% glucuronidation. Isoflavones were predominantly distributed in glandular (60%) and adipose (40%) tissue. The mean estrogen receptor β-derived 17β-estradiol equivalents were 21- to 40-fold higher in adipose and glandular tissues compared to 17β-estradiol levels. These findings suggest that isoflavones are bioavailable in breast tissue and may have stronger estrogenic effects than 17β-estradiol, potentially influencing breast tissue health [63].

A phase I clinical trial evaluated the safety, pharmacokinetics, and potential efficacy of high-dose isoflavones (genistein and daidzein) in men with prostate cancer. Twenty participants, aged 40 and older with stage B, C, or D adenocarcinoma of the prostate, received a soy isoflavone formulation delivering approximately 300 or 600 mg/day of genistein and half as much daidzein for 84 days. Chronic isoflavone treatment was associated with minimal side effects, including mild estrogenic effects such as breast changes and increased frequency of hot flashes. Pharmacokinetic analyses revealed rapid clearance of genistein and daidzein from plasma, with urinary excretion as the primary elimination route. Chronic dosing showed pharmacokinetic patterns similar to those observed with single-dose administration, with a slightly prolonged circulation time for daidzein under chronic conditions. These findings suggest that high-dose isoflavone treatment is safe and well tolerated over three months in men with prostate cancer [64].

In a randomized, placebo-controlled trial, 40 men with prostate cancer scheduled for radical prostatectomy were assigned to receive either 240 mg of clover-derived phytoestrogens or a placebo daily for two weeks before surgery. Phytoestrogen concentrations were measured in plasma and prostate tissue using time-resolved fluoroimmunoassay (TR-FIA). Baseline levels were low in all participants, with only 35% having detectable plasma equol concentrations. Supplementation resulted in a significant increase in prostate tissue phytoestrogen concentrations, with genistein levels rising by 23-fold and daidzein levels by 7-fold. Notably, prostate tissue concentrations of genistein and daidzein were more than twice as high as corresponding plasma levels in supplemented patients. Interestingly, even in the placebo group, prostate tissue concentrations of both genistein and daidzein were approximately two times higher than in plasma, suggesting that the prostate can concentrate available phytoestrogens. Additionally, 90% of patients in the phytoestrogen group showed detectable plasma equol concentrations after supplementation. These findings suggest that short-term dietary phytoestrogen supplementation significantly elevates intraprostatic concentrations of genistein and daidzein, which may have implications for prostate cancer prevention and treatment [65].

In another study, twelve men with benign prostatic hyperplasia received soy extract supplementation (3 Evestrel capsules, providing 112.5 mg isoflavone aglycone equivalents per day) for 3 days before prostate surgery. Blood and prostate tissue samples were collected, and metabolites were identified using liquid chromatography–electrospray ionization tandem mass spectrometry (LC-ESI-MS/MS) along with chemically synthesized standards of glucuronilated isoflavones. The primary metabolites found in both plasma and prostate tissue were two monoglucuronides of daidzein and two monoglucuronides of genistein. Total isoflavone concentration in prostate tissue was measured at 1.05 ± 0.62 nmol/g (range 0.30–2.23) 12 h after the final dose of isoflavone supplementation. At this time point, prostate tissue concentrations of both daidzein (0.47 nmol/g in prostate vs. 0.66 μmol in plasma) and genistein (0.58 nmol/g in prostate vs. 0.78 μmol in plasma) were found to be lower than their respective plasma concentrations [66].

A randomized, two-phase crossover pharmacokinetic study compared the bioavailability and pharmacokinetic parameters of plasma isoflavones after a single dose of soy beverage and soy extract capsules in twelve postmenopausal Thai women. Participants consumed either two soy extract capsules (containing 7.79 mg daidzin and 22.57 mg genistin) or a soy beverage made from 15 g of soy flour (containing 9.27 mg daidzin and 10.51 mg genistin) during the first phase. After a washout period of at least one week, they received the alternate preparation in the second phase. Blood samples collected up to 32 h post-administration were analyzed for plasma daidzein and genistein concentrations using HPLC, and pharmacokinetic parameters were estimated using noncompartmental analysis. The bioavailability of genistein, as assessed by dose-adjusted AUC, C_max_, and other parameters, showed no significant differences between the two preparations. Similarly, for daidzein, C_max_/dose, time to reach C_max_ (T_max_), and half-life (t_1/2_) were comparable. However, the dose-adjusted AUC for daidzein was slightly but significantly higher for soy extract capsules than for soy beverage. These findings suggest that the bioavailability of genistein is comparable between soy beverage and soy extract capsules, while the bioavailability of daidzein is marginally greater with soy extract capsules. The pharmacokinetic profiles of both isoflavones, including C_max_ and T_max_, were otherwise similar between the two delivery methods, indicating both preparations are effective sources of isoflavones for postmenopausal women [67].

The objective of a consecutive investigation was to examine the pharmacokinetics of isoflavone concentrations over 24 h in healthy adults consuming either soy foods or soy isoflavone tablets at varying doses. This randomized, cross-over trial included twelve generally healthy adults and consisted of three phases: (1) isoflavone tablets at 144 mg/day, (2) isoflavone tablets at 288 mg/day, and (3) soy foods providing a calculated 96 mg isoflavones/day (doses in aglycone equivalents), with doses divided across three meals per day. After 6 days in each phase, plasma isoflavone concentrations were measured on the seventh day at 0, 4, 8, 10, 12, and 24 h. The results indicated that average total isoflavone concentrations at 8, 10, and 12 h exceeded 4 µmol/L for both the soy food and higher-dose tablet phases. Genistein concentrations were higher in the soy food phase compared to both the lower and higher dose supplement phases. When comparing plasma concentrations for the two tablet doses, saturation appeared more pronounced for genistein than for daidzein at the higher dose. In conclusion, significant differences in the pharmacokinetics of genistein and daidzein were observed, emphasizing the influence of isoflavone source and dose on their bioavailability, with soy foods potentially offering an advantage over isoflavone supplements in increasing serum concentrations of these compounds [68].

The next study aimed to explore whether chronic intake, ethnic origin, and dietary context influence the bioavailability of soya phytoestrogens. Two prospective trials were conducted to assess the pharmacokinetics of soya-based cheese (containing 45.97 ± 1.57 mg of isoflavones) after acute and chronic intake over 10 days. Twelve healthy young Asians, who had been residing in France for 2 months, were randomized in a cross-over design to compare the effects of a Western versus an Asian dietary context. The second trial compared these Asians on a Western diet to twelve healthy young male Caucasians consuming the same diet. All participants were non-equol producers. The results showed that, after acute intake, Asians exhibited higher Cmax and AUC for both genistein and daidzein than Caucasians. In Caucasians consuming the Western diet, both Cmax and AUC values for isoflavones significantly increased after chronic intake, a trend not observed for daidzein in Asians regardless of dietary context. This study provides the first evidence that Asians have superior absorption of soya phytoestrogens compared to Caucasians following acute soya intake, independent of whether the diet is Western or Asian. Furthermore, while chronic ingestion increased the AUC and Cmax for both daidzein and genistein in Caucasians, no such changes were observed in Asians. These findings highlighted important ethnic differences in isoflavone pharmacokinetics and bioavailability, which may influence the health outcomes associated with soya consumption [69].

In a randomized, open-label, sequential-group study, 40 healthy volunteers received single oral doses of 30, 60, 150, or 300 mg of synthetic genistein aglycone. Tolerability was excellent across all doses, with no clinically significant effects observed in vital signs or laboratory parameters. Genistein demonstrated rapid absorption, with a one-peak plasma concentration-time profile. C_max_ concentrations were dose-dependent, with mean values of 252.0, 605.0, 1518.0, and 1808.0 ng/mL reached between 4.0 and 6.0 h post-administration. The t_1/2_ ranged from 7.5 to 10.2 h, and the AUC increased with dose, showing values of 2761.8, 8022.3, 21,655.0, and 27,537.8 ng·h/mL for the 30, 60, 150, and 300 mg doses, respectively. Linear regression analysis of dose-normalized AUCs supported dose linearity for the extent of absorption across the 30–300 mg range. However, C_max_ linearity was evident only up to 150 mg, suggesting limited intestinal absorption at the highest dose. These results demonstrate that synthetic genistein is safe and well tolerated at doses up to 300 mg, with nearly dose-linear pharmacokinetics for both absorption extent and rate, except at the highest dose where absorption appeared to plateau. These findings support the potential of genistein as a safe therapeutic option for postmenopausal health management [70].

Bonistein^TM^ is a synthetic genistein product with over 99.5% purity. To assess its safety, tolerability, and pharmacokinetics, a randomized, open-label, sequential-group phase I study was conducted with 30 healthy volunteers. Participants were administered 30, 60, or 120 mg of bonistein^TM^ once daily for 14 days. Blood samples were collected on day 1 (after the first dose) and day 14 (at steady state) to determine the pharmacokinetic profile. Bonistein^TM^ was well tolerated, with 33 reported adverse events, most of which were mild. No significant changes were observed in clinical laboratory values or vital signs. Pharmacokinetic analysis indicated that both the extent (AUC) and rate (Cmax) of absorption of bonistein^TM^ increased in proportion to the dose on both day 1 and day 14, suggesting that repeated dosing does not alter its absorption characteristics. These results support the safety and dose-dependent pharmacokinetic profile of bonistein^TM^ [71].

Bonistein^TM^ was also investigated in twelve healthy postmenopausal women to assess its safety, tolerability, and pharmacokinetic profile after 7 days of repeated intake. The pharmacokinetic data were collected after both the first oral dose and after 7 days of repeated daily intakes of 30 mg of the test formulation. Plasma levels of genistein (aglycone) and its conjugates were measured using a standardized LC/MS analytical method with D4-genistein as the internal standard. The plasma concentration-time profiles for conjugated genistein showed a rapid, monophasic one-peak curve reaching the C_max_ at approximately 5.9 h after the first dose and 5.3 h at steady state. The Cmax values were 456.8 ng/mL after the first dose and 498.5 ng/mL at steady state. The t1/2 was calculated as 10.8 h for the first dose and 8.2 h at steady state. The AUC for the first dose was 3949.1 h·ng/mL, and the AUC from 0 to 24 h at steady state was 5923.3 h·ng/mL. Steady-state was achieved within 4 to 5 days, with no significant accumulation observed. The test formulation was found to be safe and very well tolerated by the participants [72].

The effect of probiotic bacteria on genistein bioavailability has garnered significant interest due to the potential role of gut microbiota in modulating the absorption and metabolism of isoflavones. Probiotics, which are live microorganisms that confer health benefits to the host, can influence gut microflora balance and enzymatic activity, potentially enhancing isoflavone bioavailability. Studies suggest that probiotics may alter the composition of the gut microbiota, leading to increased conversion of isoflavones like genistein from their glucoside form to the aglycone form, which may be more readily absorbed by the intestines. Furthermore, probiotic bacteria have been shown to interact with gut enzymes, such as β-glucosidases, which hydrolyze glucoside conjugates to release bioactive aglycones. This interaction may contribute to the interindividual variation in isoflavone absorption observed after soy intake, possibly increasing the bioavailability of genistein in certain populations. Additionally, the metabolism of malonyl- and acetyl-genistin is an important aspect of genistein bioavailability and its subsequent pharmacokinetic profile in human and animal models. Studies have shown that these esterified forms of genistein are rapidly hydrolyzed in the gastrointestinal tract by the intestinal microflora, releasing the aglycone genistein, which then undergoes further metabolism. In particular, the gut microbiota possesses enzymes capable of degrading malonyl and acetyl groups, which is essential for the efficient absorption of genistein. Once released, genistein undergoes glucuronidation and sulfatization processes, mainly in the liver and intestinal cells, leading to the formation of genistein glucuronides and sulfates, which predominate in systemic circulation. Studies in animal models such as rats have confirmed similar metabolic pathways, suggesting the conservativeness of these processes between species. Furthermore, the presence of malonyl and acetyl groups may affect the rate and efficiency of these transformations, potentially modifying the bioavailability and biological activity of genistein. While the exact mechanisms remain under investigation, the modulation of gut microbial composition through probiotic supplementation presents a promising avenue for enhancing the bioavailability and efficacy of genistein, with potential implications for its health benefits, particularly concerning estrogen-related conditions [73,74,75].

Tsangalis et al. examined the effect of consuming isoflavone aglycone-enriched soy milk, fermented with *bifidobacteria*, on urinary isoflavone excretion and percentage recovery in postmenopausal women. Sixteen participants were randomly assigned to two groups in a double-blind, crossover study design, with three 14-day supplementation periods separated by 14-day washout phases. Participants consumed either fermented or non-fermented soy milk in daily doses of 20, 40, or 80 mg isoflavones, and four 24 h pooled urine samples were collected during each supplementation period. Isoflavone concentrations were analyzed via HPLC, revealing that fermented soy milk had significantly higher aglycone content (36–69%) compared to non-fermented milk (7–10%) at all doses. A linear dose–response relationship was observed for fermented soy milk, indicating less interindividual variation in isoflavone absorption compared to non-fermented milk. A trend towards a higher percentage of urinary recovery of daidzein and glycitein was noted with fermented soy milk at the 40 mg dose [76].

In another clinical trial, thirty-one participants were administered either probiotic capsules containing *Lactobacillus acidophilus* and *Bifidobacterium longum*, or placebo capsules, for 2 months. Fasting plasma concentrations of testosterone, dihydrotestosterone, androstanediol glucuronide, androstenedione, dehydroepiandrosterone sulfate, sex hormone-binding globulin (SHBG), and leptin were measured on days 1 and 57. Urinary excretion of genistein, glycitein, daidzein, O-desmethylangolensin (O-DMA), and equol was assessed on days 4 and 61 following a 4-day soy challenge. The results showed that probiotic consumption did not significantly alter equol excreter status or plasma hormone and leptin concentrations. At baseline, no differences in plasma hormone concentrations were observed between equol excreters and nonexcreters, although the small number of equol excreters in the study limited the statistical power of this finding. In conclusion, the 2-month probiotic intervention did not significantly impact equol excretion or plasma hormone and leptin concentrations. Additionally, male equol excreters in this study did not exhibit a hormone profile indicative of reduced prostate cancer risk, although this conclusion should be considered with caution [77].

A subsequent study aimed to investigate the impact of consuming bioactive yogurt (a probiotic) or resistant starch (a prebiotic) in combination with a high-soy diet on soy isoflavone bioavailability. Using a crossover design, this study involved thirty-one older males and postmenopausal females, comparing chronic soy consumption with soy plus probiotic yogurt or resistant starch over three 5-week dietary periods. Isoflavone bioavailability was assessed by analyzing plasma and urine samples before and after a standardized soy meal at the start and end of each period. The results showed no significant changes in plasma or urinary isoflavone levels following chronic soy intake, nor did the probiotic yogurt or resistant starch treatments yield notable effects. However, trends were observed for increased plasma concentrations of daidzein and genistein after probiotic treatment, and higher plasma daidzein and genistein concentrations 24 h after soy intake in participants who consumed resistant starch. Although neither treatment led to increased equol production, a trend for elevated plasma equol was observed in “equol-positive” participants after probiotic intake. Overall, the weak or absent effects of probiotic yogurt or resistant starch supplementation suggest that these interventions did not significantly alter gut microbiota in a manner that would meaningfully impact isoflavone bioavailability or metabolism [78].

In the next study, the single-dose bioavailability of two oral formulations of soy isoflavones in menopausal women undergoing antibiotic therapy, with a particular focus on the potential impact of *Lactobacillus sporogenes* on isoflavone absorption, was assessed. Twelve menopausal women participated in a controlled cross-over design study. The two treatments compared were the reference formulation (R) containing 60 mg of soy isoflavones (30 mg genistin and 30 mg daidzin), calcium, and vitamin D3, and the experimental formulation (E), which included the same components as R with the addition of 500 million spores of *Lactobacillus sporogenes*. The participants were treated with amoxicillin + clavulanate for 5 days, with a 2-week washout period between treatments. Plasma samples were collected over 24 h post-dose, and genistein and daidzein concentrations were analyzed by HPLC. The results indicated that the pharmacokinetic parameters for genistein were significantly higher after E administration compared to R, with a 24.3% increase in peak Cmax, a 24.4% increase in the AUC_0–24_, and an 11% longer mean residence time. In contrast, daidzein showed greater variability in its Cmax and AUC values with the R formulation, suggesting a more consistent absorption pattern in the formulation containing lactobacilli. These findings suggest a trend toward enhanced absorption of genistein when soy isoflavones are combined with *Lactobacillus sporogenes*, which may have implications for improving the bioavailability of soy isoflavones, especially in populations undergoing antibiotic treatment. This effect could be of particular relevance for menopausal women seeking to maximize the potential health benefits of soy isoflavones [79].

The chemical form of genistein appears to play a significant role in their absorption and plasma disposition, which can influence their biological activity. The consecutive study aimed to investigate the pharmacokinetic profiles of genistein and its phase II metabolites in the plasma and urine of healthy young women after the ingestion of multiple doses of both pure aglycone and glucoside forms of genistein. The metabolites observed in human plasma included genistein-7-glucuronide (G-7-G), 4′-glucuronide (G-4′-G), 7-sulfate (G-7-S), 4′-sulfate (G-4′-S), 4′,7-diglucuronide (G-4′,7-diG), and 7-glucuronide-4′-sulfate (G-7-G-4′-S), in addition to the unconjugated genistein. Notably, G-4′,7-diG, and G-7-G-4′-S were the predominant metabolites, comprising around 30% of the total genistein in plasma. Compared to the aglycone form, the glucoside form led to a higher concentration of total genistein in vivo, with an increased presence of the major metabolites G-4′,7-diG and G-7-G-4′-S in plasma. However, no significant differences in urinary excretion were observed between the aglycone and glucoside forms. These findings suggest that the glucoside form of genistein enhanced the absorption and plasma disposition of genistein, potentially affecting its bioavailability and subsequent biological effects. This highlights the importance of considering the chemical form of isoflavones when evaluating their health benefits, as the presence and concentration of specific metabolites in plasma may influence their pharmacological activity [80].

In a randomized cross-over trial, twelve healthy young volunteers were administered a single dose of 30 mg genistein in three different formulations: as a genistein tablet, a genistein tablet in low-fat milk, and soy milk containing genistein glycosides. High-mass resolution LC-LTQ-Orbitrap Fourier transform mass spectrometry (FTMS) was used to detect and quantify free genistein, seven of its phase-II metabolites, and fifteen gut-derived metabolites in human plasma. A novel metabolite, genistein-4′-glucuronide-7-sulfate (G-4′G-7S), was identified. Nutrikinetic analysis, using population-based modeling, revealed a consistent order of appearance for the five major genistein phase II metabolites in plasma: (1) G-4′,7-diG, (2) G-7-S, (3) G-4′,7-diG, (4) G-4′-G, and (5) G-7-G, regardless of the food matrix. These findings indicate that the conjugated genistein metabolites appear in a distinct sequence in human plasma. Notably, the early appearance of G-4′,7-diG suggests a multistep formation process for both mono- and hetero-genistein conjugates, likely involving one or two deglucuronidation steps [81].

The impact of genistein on drug-metabolizing enzymes is an important consideration for understanding its broader pharmacokinetic and pharmacodynamic effects. A study involving two weeks of daily intake of 1 g genistein in healthy Chinese female volunteers demonstrated notable changes in enzyme activity, including a decrease in cytochrome P450 (CYP1A2) and xanthine oxidase (XO) activity, alongside an increase in CYP2A6 activity. These findings suggest that genistein may modulate the activity of specific cytochrome P450 enzymes and other metabolic pathways. However, the activity of N-acetyltransferase 2 (NAT2) remained unchanged, indicating that the effect of genistein may be selective, influencing certain metabolic enzymes while sparing others. This selective modulation of enzyme activity is crucial because it implies that the pharmacokinetics of drugs metabolized by CYP1A2, XO, and CYP2A6 could be altered in individuals consuming genistein, potentially affecting the absorption, distribution, metabolism, and excretion of these drugs. The relevance of these findings extends to personalized medicine, as individuals with different dietary habits or genetic profiles may experience varying degrees of enzymatic alteration in response to genistein, influencing their response to other medications. Further research is needed to explore how genistein and similar phytoestrogens interact with a wider range of metabolic pathways and substrates, providing more comprehensive insights into their effects on drug metabolism and health outcomes [82].

The results of another study provide important insights into the effects of genistein on CYP3A and P-glycoprotein (P-gp) activity, which are key players in drug metabolism and transport. The co-administration of genistein led to a significant decrease in the pharmacokinetic parameters of the probe substrates midazolam (CYP3A) and talinolol (P-gp), indicating that genistein may induce the activity of both CYP3A and P-gp. Specifically, genistein administration resulted in a reduction in the AUC and Cmax of both midazolam and talinolol, suggesting enhanced clearance and a potential decrease in the bioavailability of drugs metabolized by CYP3A and transported by P-gp. Moreover, the increased oral clearance of midazolam and talinolol after genistein administration further supports the hypothesis that genistein may induce CYP3A and P-gp function. These findings highlight the potential of genistein to modulate drug pharmacokinetics through the induction of important metabolic enzymes and transporters, which could alter the absorption, distribution, and elimination of various drugs. While the changes observed were modest, this study underscored the need for further investigation into the clinical significance of these effects, particularly in individuals who consume genistein-rich foods or supplements, as it may influence the efficacy and safety of co-administered medications. The potential for genistein to interact with other drugs via CYP3A and P-gp induction warrants caution, especially in the context of polypharmacy or in patients with conditions requiring precise drug dosing [83].

### 4.2. Safety

The safety and pharmacokinetics of purified unconjugated isoflavones (genistein, daidzein, and glycitein) were assessed in a single-dose study involving twenty-four healthy postmenopausal women. Participants received one of two purified isoflavone formulations at doses delivering genistein at 2, 4, 8, or 16 mg/kg body weight, exceeding levels typically obtained through dietary intake. Safety assessments were conducted at intervals over 30 days, and pharmacokinetics were analyzed during the first 24 h. A 7% reduction in systolic and diastolic blood pressure and a 32% decrease in neutrophil count were observed 24 h following the administration of formulation A. Isolated incidents of nausea, pedal edema, and breast tenderness were reported, although these were considered potentially related to the treatment. Pharmacokinetic analysis revealed terminal plasma half-lives of 3.8, 7.7, and 3.4 h for free genistein, daidzein, and glycitein, respectively, with corresponding pseudo-half-lives for total genistein and daidzein of 10.1 and 10.8 h. The bioavailability of total genistein and daidzein was similar between the two formulations. These findings indicate that single high doses of purified isoflavones result in minimal clinical toxicity in postmenopausal women and suggest that chronic administration at 12–24 h intervals is unlikely to lead to significant bioaccumulation [84].

Genistein has raised concerns due to its ability to induce genetic damage [85]. A study by Miltyk evaluated the genotoxicity of a purified soy unconjugated isoflavone mixture in men with prostate cancer. Twenty patients received 300 mg of genistein daily for 28 days, followed by 600 mg daily for 56 days. Genotoxicity markers, including DNA strand breaks, chromosomal damage, and *MLL* gene translocations, were assessed in peripheral lymphocytes. Six healthy men served as controls. The results indicated no significant changes in the average or individual nuclear tail moment or micronucleus frequency (MF) across the study group, except for one subject whose MF exceeded the pre-set clinical threshold. Interestingly, a significant decrease in the average nuclear tail moment was observed at day 28 relative to baseline, suggesting a potential protective effect. No genistein-induced rearrangements of the *MLL* gene were detected in three subjects evaluated with fluorescence in situ hybridization. In vitro, genistein induced chromosomal damage at concentrations ≥100 µmol/L; however, peak plasma concentrations of total genistein and unconjugated genistein in vivo remained below 27.1 µmol/L and 0.32 µmol/L, respectively. These findings suggested that, despite in vitro genotoxic potential, therapeutic doses of genistein do not induce genetic damage in men with prostate cancer, supporting its safety in this context [86].

Similar conclusions were reported in another phase I double-blind clinical trial assessing the safety and effects of a high oral dose of soy isoflavones (900 mg/day) administered daily for 84 days in healthy postmenopausal women. The primary outcomes measured included DNA damage, apoptosis, and markers of estrogenic stimulation. After determining eligibility and equol producer status, participants were randomly assigned to either the isoflavone (active) or placebo group, with 30 women completing the study and 18 in the active group. DNA damage was assessed using a comet assay and apurinic/apyrimidinic site assays in lymphocytes, apoptosis was evaluated through terminal deoxynucleotidyl transferase-mediated dUTP nick end labeling and activated caspase-3 assays, and estrogenic effects were measured by self-report questionnaires and blood assays for estrogen, follicle-stimulating hormone (FSH), luteinizing hormone (LH), and sex hormone-binding globulin (SHBG). The results demonstrated no evidence that high doses of soy isoflavones induced DNA strand breaks, increased the abundance of apurinic/apyrimidinic sites, or enhanced apoptosis in peripheral lymphocytes. There were no significant changes in the markers of estrogenic activity or other laboratory parameters, and adverse events were minimal, limited to mild (grade 1) drug-related events. Overall, this study concluded that unconjugated soy isoflavones at a dose of 900 mg/day are safe and well tolerated in healthy postmenopausal women, with no significant adverse effects or indications of DNA damage or increased apoptosis [87].

**Figure 3:**
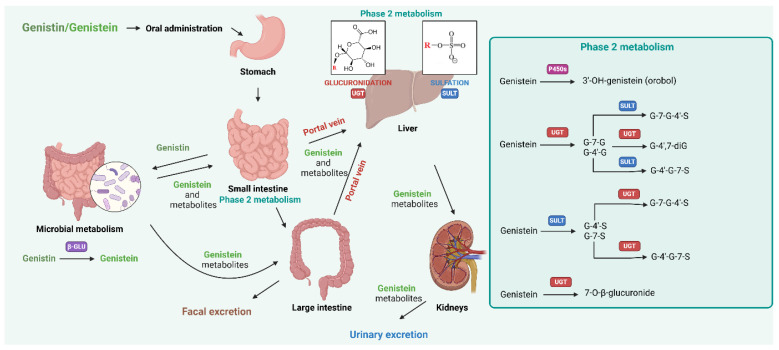
Genistin and genistein metabolism following oral consumption. Genistein metabolism in the human body involves several key processes, primarily occurring in the gastrointestinal tract and liver. After ingestion, genistein is typically absorbed in the small intestine, where it undergoes phase I metabolism, involving oxidation, reduction, or hydrolysis. Enzymes such as beta-glucosidases (β-GLU) play a crucial role in hydrolyzing genistin, the glycosylated form, into genistein, the aglycone form. Once absorbed, genistein undergoes phase II metabolism in the liver, where it is conjugated with glucuronic acid or sulfate through the actions of UDP-glucuronosyltransferases (UGTs) and sulfotransferases (SULTs), forming glucuronides and sulfates. In addition to the liver, the intestinal mucosa contains UGTs and SULTs that contribute to the conjugation of genistein. These conjugated metabolites are then circulated in the bloodstream or excreted via bile and urine. The metabolic transformations significantly affect the bioavailability and biological activity of genistein, influencing its potential health benefits. For abbreviation details, see the Abbreviations Section. Created in BioRender. Kciuk, M. (2025) https://BioRender.com/h28m531 (accessed on 17 January 2025).

The studies investigating genistein bioavailability and safety are summarized in Table 2, while the schematic representation of genistein metabolism is provided in Figure 3.

### 4.3. Novel Formulations of Genistein

Enhancing genistein bioavailability is critical to maximizing its therapeutic benefits. Formulations designed to improve solubility, stability, and absorption—such as nanoparticles and liposomes—have shown promise in overcoming these challenges (Table 3). Most of the studies employed genistein in nanoformulations for the treatment of cancer [88,89,90].

For example, nanometric porous metal–organic frameworks (nanoMOFs) have garnered significant attention as promising candidates for drug delivery applications due to their tunable structures, high surface areas, and favorable biocompatibility. Among these, the mesoporous MIL-100(Fe) framework exhibits a unique combination of characteristics that make it particularly suitable as a drug nanocarrier, including its extensive porosity, iron-based biocompatible composition, and the ability to be synthesized as stable, homogeneous nanoparticles. Recent investigations have demonstrated the potential of MIL-100(Fe) through the encapsulation of genistein via simple impregnation methods, achieving a high drug loading capacity of 27.1 wt%. A detailed understanding of the interaction between genistein and MIL-100(Fe) was obtained by integrating experimental approaches with computational modeling, which facilitated the optimization of the encapsulation process. The drug release kinetics of genistein from MIL-100(Fe) under simulated physiological conditions exhibited a sustained release profile for 3 days, suggesting the framework’s capability for controlled release and prolonged therapeutic effects. Additionally, pharmacokinetic and biodistribution studies conducted in a murine model following the oral administration of genistein-loaded MIL-100(Fe) nanoparticles indicated enhanced the bioavailability of genistein compared to free drug formulations. These results highlight the potential of MIL-100(Fe) as a versatile and effective drug delivery system, offering advantages in terms of drug loading, sustained release, and oral bioavailability. However, further investigation is required to evaluate the long-term safety, immunogenicity, and scalability of this nanoformulation to facilitate its translation into clinical applications [91].

Iqba et al. synthesized genistein nanocrystals using wet ball milling. The formulation was adjusted with Box–Behnken Design Expert to assess the effects of stabilizer concentration, drug concentration, and the number of zirconium beads (milling media) on nanoparticle size, polydispersity, and zeta potential. The NCs were surface-modified with transferrin (Tf) to create Tf-modified genistein nanoparticles to enhance cancer cell selectivity and cytotoxicity. In vivo pharmacokinetic experiments in mice following intraperitoneal injection demonstrated that the Cmax of nanocrystal formulation was 2.5 times greater than that of free genistein. The area under the curve from the time of administration to 24 h was 2.5 to 3 times greater compared to the unprocessed medication [92].

Liposomal formulations, such as conventional and stealth liposomes, incorporating unsaturated phospholipids and cholesterol, have demonstrated remarkable improvements in genistein’s solubility, stability, and release profile. Specifically, these liposomes solubilized genistein over 350-fold more effectively than aqueous drug solutions and exhibited a controlled, extended release of the drug. The enhanced cellular delivery of genistein was evidenced by the preservation of its anti-oxidant activity, which was confirmed through peroxide neutralization assays. Furthermore, liposomal genistein exhibited significant broad-spectrum anti-cancer efficacy across murine and human cancer cell lines, with reduced IC_50_ values compared to free drug controls [93].

In addition, the incorporation of genistein into liposomal systems composed of asolectin lipids has further enhanced its therapeutic potential. Studies investigating the interaction between genistein and liposomal phospholipids revealed that genistein influences lipid hydration and mobility, particularly affecting the lipid polar head and interfacial carbonyl groups, leading to improved lipid organization and decreased lipid peroxidation [99]. Moreover, phospholipid vesicles composed of 1,2-dipalmitoyl-sn-glycero-3-phosphocholine (DPPC) and 1,2-dioleoyl-sn-glycerophosphocholine (DOPC) demonstrated enhanced genistein solubilization and anti-oxidant capacity, with DOPC vesicles achieving optimal solubilization, thereby improving the bioavailability and activity of the drug [94].

Furthermore, surface-modified genistein phytosomes, such as genistein-pegylated hyaluophytosomes and genistein hyaluophytosomes, have shown promising improvements in bioavailability and therapeutic efficacy, particularly in the context of breast cancer treatment. These formulations exhibited increased particle size and zeta potential, as well as enhanced stability and controlled release compared to free genistein. The bioavailability of these formulations was significantly improved, as evidenced by higher AUC values compared to genistein suspension [95].

A novel microgel oral delivery system loaded with liposome nanoparticles (Li NPs) containing genistein was developed to treat ulcerative colitis. This system significantly enhanced the bioavailability of genistein by ensuring its controlled release in the colonic region, minimizing degradation in the harsh gastrointestinal environment. The encapsulation of genistein Li NPs in alginate microgels not only improved the drug’s stability and retention but also promoted targeted release in the colon, leading to improved therapeutic outcomes in ulcerative colitis animal models [96].

Similarly, in breast cancer therapy, the use of chitosan-coated nanoliposomes for the co-administration of poorly soluble lipophilic drugs like exemestane and genistein enhanced bioavailability by improving the drug solubility, stability, and cellular uptake of genistein. The chitosan coating facilitated better permeation and sustained release, contributing to improved therapeutic efficacy and reduced side effects compared to traditional formulations [97].

Furthermore, in lung cancer treatment, a biomimetic delivery system utilizing platelet membrane-coated liposomes has been shown to improve the bioavailability of genistein by enhancing its stability and targeting ability. This system provides slow, sustained release of the drug, ensuring prolonged exposure at the tumor site and better therapeutic outcomes [98].

These innovative delivery systems represent a promising approach to improving the bioavailability and clinical efficacy of drugs, particularly for the treatment of complex diseases such as UC, cancer, and possibly diabetes that require precise targeting and controlled release.

## 5. Critical Assessment, Prospects, and Conclusions

### 5.1. Summary of Key Findings

In conclusion, the above-mentioned studies suggest promising roles for various dietary compounds, particularly isoflavones (like genistein), anti-oxidants, and phenolic compounds, in managing metabolic dysfunctions and related diseases, such as T2DM, obesity, and cardiovascular risk. These compounds have demonstrated significant effects on metabolic parameters such as insulin resistance, blood glucose levels, lipid profiles, and inflammation. Several studies found that genistein notably reduced insulin resistance and improved blood glucose control in both postmenopausal women and individuals with obesity or T2DM. Additionally, anti-oxidant treatments, such as α-lipoic acid and vitamin combinations, showed protective effects on retinal cells in diabetic retinopathy, suggesting broader applications in diabetes management. Phenolic compounds, especially dihydrocaffeic acid and genistein diglucuronide, were identified as biomarkers linked to a lower risk of T2DM, further underlining the potential of dietary polyphenols in T2DM prevention. Moreover, the studies consistently emphasized the impact of gut microbiota modulation in improving insulin sensitivity and reducing metabolic endotoxemia, which can be facilitated by genistein. However, while the results are promising, they point to the need for further exploration of the long-term effects of these compounds, particularly in large, diverse populations.

### 5.2. The Importance of Personalized Supplementation

Additionally, variations in individual responses to these compounds were observed, indicating the importance of personalized nutrition strategies. The differential responses to isoflavones, anti-oxidants, and phenolic compounds suggest that personalized approaches to nutrition could be more effective than one-size-fits-all solutions. Understanding genetic, microbiota, and lifestyle factors that influence individual responses could pave the way for tailored dietary interventions in managing T2DM, obesity, and related metabolic disorders. The modulation of gut microbiota by dietary compounds such as genistein presents an exciting avenue for future research. Understanding the specific microbial changes that mediate metabolic improvements, particularly insulin resistance, could lead to more targeted and effective interventions. While short-term studies have shown positive results, the long-term safety and efficacy of these compounds in diverse populations, including those with comorbidities, remain unclear.

### 5.3. Future Research Directions and Challenges

Future studies should address the potential side effects and long-term health benefits or risks of sustained consumption of isoflavones and other dietary compounds. Moving from experimental models and small clinical trials to large-scale clinical applications is a critical step. More extensive trials in both diabetic and non-diabetic populations, especially in clinical settings, will be necessary to validate the therapeutic potential of these dietary interventions and to determine the optimal dosing, formulation, and duration of use. Currently, there are only two completed clinical trials (NCT00951912 and NCT01302639 registered on (https://clinicaltrials.gov/search?cond=Type%20II%20Diabetes%20Mellitus&intr=Genistein accessed on 17 January 2025)) that mention genistein use in T2DM. While the studies show promising results in terms of outcomes like insulin resistance and glucose control, the exact mechanisms by which genistein exerts these effects remain unclear. Detailed mechanistic studies are necessary to understand how genistein and related compounds interact with metabolic pathways, gut microbiota, and inflammatory processes. Many of the already performed clinical studies involved specific groups, such as postmenopausal women, individuals with MetS, or those with obesity. Further research should include more diverse populations, including men, younger individuals, those from various ethnic backgrounds, and those with more severe or complex metabolic conditions, to assess the universal applicability of these interventions. The optimal dose, bioavailability, and formulation of genistein and other phenolic compounds require clarification. Studies should investigate how different dosages, forms (e.g., whole foods vs. supplements), and combinations with other nutrients affect outcomes. Additionally, assessing the long-term safety and side effects of high-dose interventions is essential. Many participants in performed clinical studies had multiple risk factors, such as high cholesterol, hypertension, and obesity. Investigating how genistein and related compounds interact with other comorbid conditions, especially cardiovascular diseases, could provide a more comprehensive understanding of their benefits. While dietary interventions with isoflavones and anti-oxidants show promise, comparative studies with conventional treatments (e.g., metformin for T2DM or statins for hyperlipidemia) will help determine whether these compounds offer comparable or additional benefits.

## Figures and Tables

**Table 1:** Clinical trials on genistein and its impact on type II diabetes mellitus (T2DM).

Study	Study Design	Participants	Intervention	Duration	Key Findings
Fanti et al. PMID: 16766544 [37]	Randomized controlled trial	HD patients with elevated systemic inflammation	Soy-based isoflavone-rich nutritional products	8 weeks	5–10× increase in serum isoflavone levels (genistein: 337.9 nM vs. 41.4 nM). Inverse correlation between Δ-isoflavone and CRP levels. Positive correlation with albumin and IGF-1. A trend toward lower CRP levels.
Charles et al. PMID: 18981951 [38]	Randomized controlled trial	75 healthy postmenopausal women	160 mg isoflavones vs. placebo	12 weeks	Significant increase in serum adiponectin. No significant changes in glucose, insulin, or inflammatory cytokines. Decreased TNF-α in the placebo group.
Nebbioso et al. PMID: 22044044 [39]	Randomized controlled trial	32 preretinopathic diabetes patients	α-lipoic acid (400 mg/day) + genistein + vitamins vs. placebo	30 days	Increased plasma anti-oxidant levels. Improved ERG oscillatory potential values. Enhanced retinal function.
Squadrtio et al. PMID: 23824420 [40]	Double-blind, placebo-controlled trial	120 postmenopausal women with MetS	54 mg genistein daily vs. placebo	1 year	Decreased fasting glucose, insulin, and HOMA-IR. Increased HDL-C (46.4 to 56.8 mg/dL). Decreased LDL-C (108.8 to 78.7 mg/dL). Reduced blood pressure. Well tolerated with no significant adverse events.
Braxas et al. PMID: 31307913 [41]	Randomized, double-blind, placebo-controlled trial	54 postmenopausal women with T2DM	54 mg genistein twice daily vs. placebo	12 weeks	Reduced FBS, A1C, triglycerides. Decreased MDA. Increased TAC and HDL-C. Improved insulin sensitivity (QUICKI). No changes in anthropometric indices.
Ruiz Esparza et al. PMID: 31759909 [42]	Randomized dietary intervention	60 healthy university students	15 g legume-based product daily	3 months	Reduced serum glucose. Decreased MDA and HOMA-IR. No changes in anthropometric measures or lipids.
Guevara- Cruz et al. PMID: 32152146 [43]	Randomized controlled trial	45 participants with obesity and insulin resistance	50 mg genistein daily vs. placebo	2 months	Reduced insulin resistance (HOMA-IR). Increased *Verrucomicrobia* in gut microbiota. Enhanced *AMPK* phosphorylation. Increased β-oxidation metabolites.
Domínguez-López et al. PMID: 37062219 [44]	A case–control study (PREDIMED trial)	T2DM patients	Analysis of urinary phenolic compounds	N/A	Genistein diglucuronide is associated with lower T2D risk. No direct effect on plasma glucose.

A1C—hemoglobin A1c; AMPK—AMP-activated protein kinase; CRP—C-reactive protein; ERG—electroretinogram; FBS—fasting blood sugar; HDL-C—high-density lipoprotein cholesterol; HD—hemodialysis; HOMA-IR—homeostatic model assessment of insulin resistance; IGF-1—insulin-like growth factor 1; LDL-C—low-density lipoprotein cholesterol; MDA—malondialdehyde; MetS—metabolic syndrome; N/A—not available: PREDIMED—prevención con dieta mediterránea; QUICKI—quantitative insulin sensitivity check index; TAC—total anti-oxidant capacity; T2DM—type 2 diabetes mellitus; TNF-α—tumor necrosis factor-alpha.

**Table 2:** The studies investigating genistein bioavailability and safety.

Title	Study Groups/Dosage Regimen	Key Findings	PMID
High-performance liquid chromatographic assay of isoflavonoids and coumestrol from human urine	Single individual/4 days; 11 individuals across two consecutive nights following administration of roasted soybeans in dosage ranges of 5–20 g and 44–96 g, respectively.	Genistein showed rapid absorption, peaking in urine within 16–24 h post-soybean intake, with ~24.8% recovery over 88 h. Minimal free genistein (<2%) indicated extensive conjugation, and kinetics correlated with dose.	7894693 [45]
Urinary isoflavonoid phytoestrogen and lignan excretion after consumption of fermented and unfermented soy products	Nine-day feeding intervals after five days of baseline data acquisition. A total of 22 subjects (17 finished the trial). Fermented soy product (112 g tempeh) or unfermented soy (125 g soybean pieces).	Soy diets increased urinary isoflavonoid excretion and decreased lignan excretion. The tempeh diet led to higher daidzein and genistein recovery than soybean pieces. Five of seventeen subjects excreted high equol, with lower O-DMA and daidzein excretion.	7722188 [46]
Vegetables, fruits, and legumes: effect on urinary isoflavonoid phytoestrogen and lignan excretion	In total, 7 men and 3 women consumed four experimental diets in an assigned random order: a controlled basal diet, a legume/allium diet (containing garbanzo beans, garlic, and onions), and diets low or high in vegetables and fruits (containing apples, pears, potatoes, and carrots) for 9 days. Urine samples that were collected while subjects consumed their habitual diets and during the last 3 days of each feeding period were analyzed.	A high vegetable/fruit diet increased enterodiol excretion, while legume/allium diet increased O-DMA, genistein, and total isoflavonoid excretion. Equol excretion was higher on basal and legume/allium diets, linked to higher milk-based pudding intake. Excretion patterns reflected dietary changes.	7797807 [47]
The bioavailability of soybean isoflavones depends upon gut microflora in women	Seven women ingested 3.4, 6.9, or 10.3 μmol of isoflavones per kilogram of body weight from soy milk during each of three meals in a liquid diet throughout three feeding days, which were separated by two-week washout intervals.	Soy milk isoflavone doses showed gut microbiota influence on genistein bioavailability, with urinary recovery ranging from 10% to 37% and plasma levels 2.5-fold higher in low fecal excreters. Genistein’s intestinal half-life was 3.3 h, reflecting rapid microbial metabolism.	7666247 [48]
Soy isoflavonoids and cancer prevention. Underlying biochemical and pharmacological issues	Healthy volunteers (3 males, 1 female) consumed two 8-ounce soy protein beverages containing 20 g of isolated soy protein daily at 8 AM and 12 noon for 14 days.	Genistein is efficiently absorbed from the gastrointestinal tract, metabolized in the liver, and excreted in bile as 7-O-beta-glucuronide, which is reabsorbed in the distal small intestine. Soy beverage intake led to plasma genistein/daidzein levels of 0.55–0.86 µmol, mostly as conjugates, indicating enterohepatic circulation.	8886128 [49]
A diet high in wheat fiber decreases the bioavailability of soybean isoflavones in a single meal fed to women	Seven healthy women were randomly allocated in a crossover design to either a control diet comprising 15 g of dietary fiber or a wheat fiber-supplemented meal having 40 g of dietary fiber, both administered with a single dose of 0.9 mg isoflavones per kg of body weight derived from tofu or texturized vegetable protein (TVP).	Dietary fiber reduced genistein bioavailability, with 55% lower plasma levels (*p* < 0.05) and 20% lower urinary excretion (*p* < 0.03). Daidzein excretion was unaffected. Tofu led to 23% higher urinary genistein than TVP (*p* < 0.02), reflecting differences in genistein content between soy foods.	8613890 [50]
Neither background diet nor type of soy food affects short-term isoflavone bioavailability in women	Eight women administered 0.9 mg/kg of isoflavones from soy milk at 0730, 1230, and 1730 h for one day. Participants consumed three backdrop diets in a random sequence: a diet prepared for them (basic foods diet), a self-selected diet at designated periods, or a self-selected diet ingested ad libitum. In subsequent research, women were administered single isoflavone dosages ranging from 0.8 to 1.4 mg/kg through breakfast casseroles including tofu, tempeh, cooked soybeans, or texturized vegetable protein.	Isoflavone bioavailability was unaffected by background diet or soy food source. Urinary daidzein recovery was higher (26–51%) than genistein (9–20%), with minimal fecal recovery due to bacterial breakdown. Plasma levels were consistent across diets (1.4 ± 0.7 µmol/L).	10736332 [51]
Urinary isoflavonoid excretion in humans is dose-dependent at low to moderate levels of soy protein consumption	Double-blind, crossover study including 14 men and women consuming 0–36 mg isoflavones daily on four 9 d diet treatment periods. Throughout each treatment phase, participants ingested a low-phytoestrogen-controlled diet alongside a beverage containing 0, 5, 10, or 20 g of soy protein. Urine gathered during the final three days of each treatment session was examined for isoflavonoid concentration.	Urinary isoflavonoid excretion showed a linear dose response to soy intake (0–36 mg isoflavones), with no differences between equol excreters and nonexcreters. Lignan excretion was unaffected by diet. Isoflavonoid excretion is dose-dependent at low to moderate soy consumption.	9209168 [52]
Pharmacokinetics of soybean isoflavones in plasma, urine, and feces of men after ingestion of 60 g baked soybean powder (kinako)	Seven men after ingestion of 60 g kinako (baked soybean powder, containing 103 micromol daidzein and 112 micromol genistein).	The plasma concentration of genistein elevated after 2 h, with a peak value of 2.44 ± 0.65 micromol/L after 6 h. Urinary excretion of daidzein was higher than genistein, and most isoflavones were recovered in feces (54.7%). Plasma half-lives were 8.36 h for genistein and 5.79 h for daidzein. Metabolite concentrations varied significantly between high and low metabolizers.	9772140 [53]
Clinical characteristics and pharmacokinetics of purified soy isoflavones: single-dose administration to healthy men	Thirty healthy males consumed a single dosage of one of two isoflavone formulations extracted from soy. The supplied doses of genistein were 1, 2, 4, 8, or 16 mg/kg body weight. Formulation A consisted of 90 ± 5% genistein, 10% daidzein, and 1% glycitein. Formulation B consisted of 43% genistein, 21% daidzein, and 2% glycitein.	No significant behavioral or physical changes were observed. Mild elevations in lipoprotein lipase and hypophosphatemia were noted without clinical toxicity. Significant amounts of isoflavones were excreted as conjugates. The elimination half-lives were 3.2 h for free genistein and 4.2 h for free daidzein, with pseudo-half-lives of 9.2 h for total genistein and 8.2 h for total daidzein.	11756070 [56]
Hydrolysis of isoflavone glycosides to aglycones by beta-glycosidase does not alter plasma and urine isoflavone pharmacokinetics in postmenopausal women	Six European, postmenopausal women ingested either of two soy beverages at one-week intervals at a concentration of 1 mg/kg body weight of total isoflavones.	Enzymatic hydrolysis of soy isoflavones to aglycones before consumption did not enhance their bioavailability. Plasma and urinary pharmacokinetics of daidzein, genistein, and glycitein were similar for both hydrolyzed and non-hydrolyzed drinks. Plasma isoflavone concentrations reached 4–5 µmol/L, and secondary metabolites were detected in plasma and urine. The ratios of isoflavones differed between food, plasma, and urine, indicating variable absorption and retention.	12221213 [58]
Safety and pharmacokinetics of purified soy isoflavones: single-dose administration to postmenopausal women	Twenty-four healthy postmenopausal women consumed a single dose of one of two pure isoflavone preparations derived from soybeans, providing a genistein dosage of 2, 4, 8, or 16 mg/kg body weight. Toxicity assessments were conducted 24 h and 3, 6, 14, and 30 days post-isoflavone treatment.	Treatment with formulation A resulted in a 7% decrease in blood pressure and a 32% decrease in neutrophil count, with mild side effects (nausea, edema, breast tenderness). The plasma half-lives for free genistein, daidzein, and glycitein averaged 3.8, 7.7, and 3.4 h, respectively. Pseudo-half-lives for total genistein and daidzein averaged 10.1 and 10.8 h. Bioavailabilities of genistein and daidzein were similar for both formulations. Minimal clinical toxicity was observed, and chronic dosing would not lead to progressive accumulation of these isoflavones.	12399289 [84]
Lack of significant genotoxicity of purified soy isoflavones (genistein, daidzein, and glycitein) in 20 patients with prostate cancer	A total of 20 patients diagnosed with prostate cancer received a treatment regimen of 300 mg of genistein per day for 28 days, followed by an increased dosage of 600 mg per day for an additional 56 days.	No DNA damage in patients’ lymphocytes was observed following genistein treatment.	12663286 [86]
Effects of a high daily dose of soy isoflavones on DNA damage, apoptosis, and estrogenic outcomes in healthy postmenopausal women: a phase I clinical trial	Thirty postmenopausal women administered daily for 84 days with capsules. Each capsule contained 150 mg of genistein activity, with the specific composition of the PTI G-2535 capsules (Lot # UPM 9809-021) being as follows: Genistein: 139.5 mg per capsule; Daidzein: 74 mg per capsule; Glycitein: 11 mg per capsule.	In treated postmenopausal women, high doses of soy isoflavones did not cause DNA strand breakage, increase in abundance of apurinic/apyrimidinic sites, or apoptosis in peripheral lymphocytes. No significant changes were observed in estrogenic effects or other laboratory measurements. Adverse events were rare, and those related to the drug were mild or grade 1 in severity.	18446090 [87]
Phase I pharmacokinetic and pharmacodynamic analysis of unconjugated soy isoflavones administered to individuals with cancer	Four patients received single doses of two different preparations of unconjugated soy isoflavones (PTI G-2535 and PTI G-4660 containing 43% and 90% genistein, respectively). Sequential cohorts received genistein at 2, 4, or 8 mg/kg orally.	One patient developed a rash, but no other toxicities were observed. Maximal plasma concentrations ranged from 4.3 to 16.3 µM for total genistein and 0.066 to 0.17 µM for free genistein. Half-life was 15.03 h for PTI G-2535 and 22.41 h for PTI G-4660, with a trend toward a higher area under the curve for PTI G-2535 (*p* = 0.07 at 8 mg/kg). Increased tyrosine phosphorylation was observed in blood cells.	14652284 [60]
Urinary isoflavone kinetics: the effect of age, gender, food matrix, and chemical composition	Twenty premenopausal women, seventeen postmenopausal women, and twenty males were administered a specified single oral bolus dose (0.44 mg isoflavones/kg body weight) of soya milk, textured vegetable protein (TVP), or tempeh on three distinct occasions. Baseline and four successive complete 24 h pooled urine samples were taken during each interval.	Urinary genistein recovery was influenced by gender and food matrix. For women, recovery was higher following soya milk consumption compared with TVP, and tempeh also led to increased urinary genistein recovery compared to soy milk in premenopausal women. No differences in urinary genistein recovery between soya foods were observed in men. While urinary daidzein excretion remained consistent across foods and unaffected by age or gender, conversion to equol, its intestinal metabolite, showed food matrix effects: urinary equol excretion was higher after tempeh ingestion among equol producers. These results suggest that genistein absorption may vary by gender and food matrix, and daidzein metabolism could be influenced by the isoflavones’ chemical composition. Further studies are needed to explore the impact of higher intake and these factors in elderly populations.	15035683 [61]
Clinical characteristics and pharmacokinetics of purified soy isoflavones: multiple-dose administration to men with prostate neoplasia	Twenty males diagnosed with stage B, C, or D adenocarcinoma of the prostate were treated with a multiple-dose regimen of a soy isoflavone formulation providing roughly 300 or 600 mg/day of genistein and half that dosage of daidzein for 84 days.	The treatment resulted in minor side effects, including some estrogenic effects (breast changes, increased frequency of hot flashes). Serum dehydroepiandrosterone decreased by 31.7% by the end of the treatment. For most subjects, prostate-specific antigen (PSA) levels had been rising before the trial, and this trend continued during the treatment, with an accelerated rate of increase after treatment ended, though this difference was not statistically significant. Genistein and daidzein were quickly cleared from plasma and excreted in urine. Pharmacokinetic data for chronic dosing were similar to those for single-dose administration, with a slightly longer circulation time observed for daidzein.	15231450 [64]
Effects of isoflavone supplements vs. soy foods on blood concentrations of genistein and daidzein in adults	The intervention comprised three phases: (1) isoflavone tablets at 144 mg/day, (2) isoflavone tablets at 288 mg/day, and (3) soy meals formulated to deliver an estimated 96 mg of isoflavones per day (doses in aglycone equivalents). Doses were distributed across three meals daily. Following six days in each experimental phase, plasma isoflavone concentrations were measured on the seventh day at 0, 4, 8, 10, 12, and 24 h. The study involved 12 healthy adults.	Average levels of total isoflavone concentrations at 8, 10, and 12 h were greater than 4 micromol/L during the soy food phase and the higher dose tablet phase. Genistein concentrations were consistently higher in the soy food phase compared to both the lower and higher dose supplement phases. When comparing plasma concentrations between the two tablet doses, saturation appeared more evident for genistein than for daidzein at the higher dose level. In conclusion, the study observed significant differences in the pharmacokinetics of genistein and daidzein depending on the source and dose of isoflavones. Notably, consuming soy foods seemed to provide an advantage in increasing serum concentrations of isoflavones compared to taking isoflavone supplements.	18602820 [68]
Safety, tolerability, and pharmacokinetics of single ascending doses of synthetic genistein (Bonistein^TM^) in healthy volunteers	Single oral dosages of 30, 60, 150, or 300 mg were given to 40 healthy participants.	Genistein was found to be safe and well tolerated in healthy volunteers, with no clinically significant effects on vital signs, ECG, or clinical laboratory parameters. Genistein was rapidly absorbed, reaching peak plasma concentrations (Cmax) after 4–6 h. Mean Cmax values increased with doses 252.0, 605.0, 1518.0, and 1808.0 ng/mL for doses of 30, 60, 150, and 300 mg, respectively. The mean terminal elimination half-lives were between 7.5 and 10.2 h. The area under the curve (AUC) values were dose-dependent, with mean AUCs ranging from 2761.8 to 27,537.8 ngxhr/mL. A linear relationship was observed between dose and AUC (extent of absorption) for doses ranging from 30 to 300 mg. However, for doses above 150 mg, the rate of absorption (Cmax) showed a plateau, indicating a limit to the intestinal absorption rate at higher doses. Genistein was safe and well tolerated, with nearly dose-linear pharmacokinetics for absorption and distribution at doses up to 150 mg.	15943224 [70]
Repeated oral once daily intake of increasing doses of the novel synthetic genistein product Bonistein^TM^ in healthy volunteers	Thirty healthy participants were administered 30, 60, or 120 mg once daily for 14 days in three successive groups. Blood samples were collected on study days 1 (post-first dose) and 14 (steady state to assess the pharmacokinetic characteristics of Bonistein^TM^).	The repeated administration of Bonistein^TM^ was well tolerated. A total of 33 adverse events were documented, predominantly of mild severity. No significant alterations in clinical laboratory results or vital signs were noted. The pharmacokinetic properties of Bonistein^TM^ demonstrated similar outcomes for the extent and rate of absorption on days 1 and 14. The AUC and Cmax values of Bonistein^TM^ escalated in direct correlation with the dosage.	16254818 [71]
Bioavailability of isoflavone phytoestrogens in postmenopausal women consuming soya milk fermented with probiotic bifidobacteria	Sixteen postmenopausal women were randomly assigned to two groups to drink either fermented or unfermented soy milk. Each group engaged in a double-blind, crossover trial consisting of three 14-day supplementation periods, interspersed with a 14-day washout phase. Participants consumed three daily doses of isoflavone through soya milk and collected four 24 h pooled urine samples during each supplementation session. Soya milk was produced using soya protein isolate and soya germ, thereafter undergoing fermentation with bifidobacteria. Isoflavone concentrations were measured by HPLC. Non-fermented soy milk with 20, 40, and 80 mg isoflavone per 200 mL contained 10%, 9%, and 7% aglycone, respectively, but their fermented equivalents contained 69%, 57%, and 36% aglycone.	A linear dose–response was observed in the fermented soya milk group suggesting more consistent absorption of isoflavones compared to the non-fermented group, which showed more interindividual variation. The total urinary isoflavone excretion was similar in both fermented and non-fermented soya milk groups. Increasing the isoflavone dosage resulted in higher urinary excretion, but the percentage of isoflavone recovered in urine decreased with higher dosages. A dosage range of 20 to 30 mg/day is suggested to be the most bioavailable source of isoflavones, whether from aglycone-rich fermented soya milk or glucoside-rich non-fermented soya milk.	16022756 [76]
Increased probiotic yogurt or resistant starch intake does not affect isoflavone bioavailability in subjects consuming a high soy diet	The bioavailability of isoflavones from soy plus probiotic yogurt or resistant starch in 31 older males and postmenopausal ladies was evaluated at the commencement and conclusion of each 5-week dietary phase by collecting plasma and urine samples following a standardized soy meal.	Chronic use of soy did not markedly influence plasma or urine isoflavones following the soy meal, nor were there substantial impacts from probiotic or resistant starch treatment. Nonetheless, there were observed tendencies indicating elevated circulating plasma daidzein and genistein following probiotic treatment, as well as increased plasma daidzein and genistein 24 h post-soy consumption with resistant starch treatment. The ineffectiveness or lack of impact of probiotic yogurt or resistant starch supplements on a chronic soy diet indicates that gut bacteria were not altered in a way that meaningfully influenced isoflavone bioavailability or metabolism.	17656069 [78]
Nutrikinetic modeling reveals the order of genistein phase II metabolites’ appearance in human plasma	Twelve healthy young volunteers received a single dosage of 30 mg of genistein in the form of a genistein tablet, a genistein tablet mixed with low-fat milk, and soy milk containing genistein glycosides.	A high-mass resolution LC-LTQ-Orbitrap FTMS platform identified and quantified in human plasma: free genistein, seven phase-II metabolites, and 15 gut-derived metabolites. A new metabolite, genistein-4′-glucuronide-7-sulfate (G-4′G-7S), was found. Nutrikinetic analysis employing population-based modeling elucidated the sequential emergence of five genistein phase II metabolites in plasma: (1) genistein-4′,7-diglucuronide, (2) genistein-7-sulfate, (3) genistein-4′-sulfate-7-glucuronide, (4) genistein-4′-glucuronide, and (5) genistein-7-glucuronide, irrespective of the food matrix.	25045152 [81]
Genistein alters caffeine exposure in healthy female volunteers	A single 100 mg dosage of caffeine was provided once before and once on the final day of a 14-day treatment regimen with 1 g of genistein daily to 18 healthy female participants. Urine and blood specimens were obtained at intervals of up to 12 and 24 h, respectively, following each caffeine administration.	After two weeks of administering 1 g of genistein daily, there were reductions in CYP1A2 and XO activity and an elevation in CYP2A6 activity, but NAT2 activity remained unchanged in healthy Chinese female participants. The pharmacokinetics of other substrates of the enzymes examined may be similarly affected.	21222115 [82]

AUC—area under the curve; C_max_—maximum concentration; CYP1A2—cytochrome P450 1A2; CYP2A6—cytochrome P450 2A6; FTMS—Fourier transform mass spectrometry; HPLC—high-performance liquid chromatography; LTQ—linear trap quadrupole; NAT2—N-acetyltransferase 2; ngxhr/mL—nanograms per hour per milliliter; O-DMA—O-desmethylangolensin; PSA—prostate-specific antigen; PTI G-2535—Protein Technologies International G-2535; TVP—texturized vegetable protein; XO—xanthine oxidase.

**Table 3:** Novel nanoformulations of genistein.

Drug Delivery System	Formulation/Method	Key Features	Bioavailability Enhancement	PMID
Nanometric porous metal–organic frameworks (NanoMOFs)	Encapsulation of genistein in mesoporous MIL-100(Fe) framework	High drug loading capacity (27.1 wt%), sustained release over 3 days, high porosity, iron-based biocompatibility, simple impregnation method	Enhanced bioavailability of genistein compared to free drug formulations, improved oral bioavailability	33596280 [91]
Genistein nanocrystals (Gen-NCs)	Wet ball milling, surface-modified with transferrin (Tf) to create Tf-Gen-NC	Adjusted formulation using Box–Behnken Design Expert, nanoparticle size, zeta potential optimization, enhanced cancer cell selectivity, cytotoxicity	Increased C_max_ (2.5×) and AUC (2.5–3×) in vivo compared to free genistein	39447935 [92]
Liposomes (GenLip)	Conventional and stealth liposomes with unsaturated phospholipids and cholesterol	Enhanced solubility (350-fold), controlled extended release, preserved anti-oxidant activity, broad-spectrum anti-cancer efficacy	Enhanced solubility, stability, and cellular delivery of genistein, increased therapeutic efficacy	24151835 [93]
Asolectin lipid-based liposomes	Liposomes composed of asolectin lipids for genistein solubilization	Interaction between genistein and liposomal phospholipids improved lipid hydration, mobility, and reduced lipid peroxidation	Improved solubilization, anti-oxidant capacity, and bioavailability of genistein	30542013 [94]
Phytosomes (G-PHA and G-HA)	Surface-modified genistein phytosomes (pegylated hyaluophytosomes and hyaluophytosomes)	Increased particle size and zeta potential, enhanced stability, controlled release	Significant improvement in bioavailability, evidenced by higher AUC compared to free genistein	36156294 [95]
Microgel oral delivery system	Liposome nanoparticles (Li NPs) containing genistein encapsulated in alginate microgels	Controlled release in the colonic region, enhanced stability, targeted drug release	Enhanced bioavailability, improved therapeutic outcomes in ulcerative colitis animal models	37877170 [96]
Chitosan-coated nanoliposomes	Nanoliposomes coated with chitosan for co-administration of exemestane and genistein	Enhanced solubility, stability, cellular uptake, sustained release	Enhanced bioavailability, improved therapeutic efficacy, and reduced side effects in breast cancer	38434864 [97]
Biomimetic liposomes (PLTM-coated)	Platelet membrane (PLTM)-coated liposomes for genistein delivery	Slow, sustained release, enhanced stability, improved tumor-targeting ability	Improved bioavailability, prolonged exposure at the tumor site, enhanced therapeutic outcomes	39430311 [98]

AUC—area under the curve; C_max_—maximum concentration; G-HA—hyaluophytosomes; G-PHA—pegylated hyaluophytosomes; GenLip—genistein liposomes; Gen-NCs—genistein nanocrystals; Li NPs—liposome nanoparticles; MIL-100(Fe)—mesoporous iron-based framework; NanoMOFs—nanometric porous metal–organic frameworks; PLTM—platelet membrane; Tf—transferrin; Tf-gen-NC—transferrin-modified genistein nanocrystals.

## Data Availability

No new data were created or analyzed in this study. Data sharing is not applicable to this article.

## Abbreviations

A1C	glycated hemoglobin
ABCC8	ATP binding cassette subfamily C member 8
AGEs	advanced glycation end-products
AKT/PKB	protein kinase B
AMPK	5′AMP-activated protein kinase
AO	oral anti-oxidant
AUC	area under the curve
BMI	body mass index
cAMP	cyclic adenosine monophosphate
C_max_	maximum concentration
CRP	C-reactive protein
CYP	cytochrome P450
DOPC	1,2-dioleoyl-sn-glycerophosphocholine
DPPC	1,2-dipalmitoyl-sn-glycero-3-phosphocholine
ER	estrogen receptor
ERG	electroretinogram
ESRD	end-stage renal disease
FBS	fasting blood glucose
FSH	follicle-stimulating hormone
FTMS	Fourier transform mass spectrometry
FTO	fat mass and obesity
G-4′,7-diG	genistein-4′,7-diglucuronide
G-4′-G	genistein-4′-glucuronide
G-4′-S	genistein-4′-sulfate
G-7-G	genistein-7-glucuronide
G-7-G-4′-S	genistein-7-glucuronide-4′-sulfate
G-7-S	genistein-7-sulfate
GLUT	glucose transporter
HD	hemodialysis
HLD-C	high-density lipoprotein cholesterol
HNF1A	hepatocyte nuclear factor-1 alpha
HOMRA-IR	homeostatic model assessment of insulin resistance
HPLC	high-performance liquid chromatography
IGF-1	insulin-like growth factor-1
IL	interleukin
IRS1	insulin receptor substrate 1
LC-ESI-MS/MS	liquid chromatography–electrospray ionization tandem mass spectrometry
LDL-C	low-density lipoprotein cholesterol
LH	luteinizing hormone
Li NPs	liposome nanoparticles
MDA	malondialdehyde
MetS	metabolic syndrome
MF	micronucleus frequency
MS	mass spectrometry
nanoMOFs	nanometric porous metal–organic frameworks
NAT2	N-acetyltransferase 2
NF-κB	nuclear factor kappa-light-chain-enhancer of activated B cells
O-DMA	O-desmethylangolensin
P-gp	P-glycoprotein
PI3K	phosphatidylinositol 3-kinase
PKA	protein kinase A
PPARG	peroxisome proliferato-activated receptor gamma
PRD	preretinopathic diabetes
PREDIMED	Prevención con Dieta Mediterránea
QUICKI	quantitative insulin sensitivity check index
SASP	senescence-associated secretory phenotype
SERM	selective estrogen receptor modulator
SHBG	sex hormone-binding globulin
SULT	sulfotransferase
T2DM	type 2 diabetes mellitus
TAC	total anti-oxidant capacity
TCF7L2	transcription factor 7 like 2
Tf	transferrin
TG	serum triglycerides
t_max_	time to maximum concentration
TNF-α	tumor necrosis factor-alpha
TR-FIA	time-resolved fluoroimmunoassay
TVP	textured vegetable protein
UC	ulcerative colitis
UGT	UDP-glucuronosyltransferase

## References

[1] Rahman U., Younas Z., Ahmad I., Yousaf T., Latif R., Rubab U., Hassan H., Shafi U., Mashwani Z.-R. (2024). Enhancing Health and Therapeutic Potential: Innovations in the Medicinal and Pharmaceutical Properties of Soy Bioactive Compounds. Front. Pharmacol..

[2] Rizzo G., Baroni L. (2018). Soy, Soy Foods and Their Role in Vegetarian Diets. Nutrients.

[3] Mateos-Aparicio I., Redondo Cuenca A., Villanueva-Suárez M.J., Zapata-Revilla M.A. (2008). Soybean, a Promising Health Source. Nutr. Hosp..

[4] Aguiar C.L., Haddad R., Eberlin M.N., Carrão-Panizzi M.C., Tsai S.M., Park Y.K. (2012). Thermal Behavior of Malonylglucoside Isoflavones in Soybean Flour Analyzed by RPHPLC/DAD and Eletrospray Ionization Mass Spectrometry. LWT-Food Sci. Technol..

[5] Garbiec E., Cielecka-Piontek J., Kowalówka M., Hołubiec M., Zalewski P. (2022). Genistein-Opportunities Related to an Interesting Molecule of Natural Origin. Molecules.

[6] Kudou S., Fleury Y., Welti D., Magnolato D., Uchida T., Kitamura K., Okubo K. (1991). Malonyl Isoflavone Glycosides in Soybean Seeds (Glycine Max Merrill). Agric. Biol. Chem..

[7] Yerramsetty V., Gallaher D.D., Ismail B. (2014). Malonylglucoside Conjugates of Isoflavones Are Much Less Bioavailable Compared with Unconjugated β-Glucosidic Forms in Rats. J. Nutr..

[8] Yang Z., Kulkarni K., Zhu W., Hu M. (2012). Bioavailability and Pharmacokinetics of Genistein: Mechanistic Studies on Its ADME. Anticancer Agents Med. Chem..

[9] Naponelli V., Piscazzi A., Mangieri D. (2025). Cellular and Molecular Mechanisms Modulated by Genistein in Cancer. Int. J. Mol. Sci..

[10] Fukutake M., Takahashi M., Ishida K., Kawamura H., Sugimura T., Wakabayashi K. (1996). Quantification of Genistein and Genistin in Soybeans and Soybean Products. Food Chem. Toxicol..

[11] Pawłowski W., Caban M., Lewandowska U. (2024). Cancer Prevention and Treatment with Polyphenols: Type IV Collagenase-Mediated Mechanisms. Cancers.

[12] Nestor M.S., Bhupalam V., Awad N., Hetzel J.D. (2024). The Therapeutic Role of Genistein in Perimenopausal and Postmenopausal Women. J. Clin. Aesthetic Dermatol..

[13] Galicia-Garcia U., Benito-Vicente A., Jebari S., Larrea-Sebal A., Siddiqi H., Uribe K.B., Ostolaza H., Martín C. (2020). Pathophysiology of Type 2 Diabetes Mellitus. Int. J. Mol. Sci..

[14] Son J., Accili D. (2023). Reversing Pancreatic β-Cell Dedifferentiation in the Treatment of Type 2 Diabetes. Exp. Mol. Med..

[15] Lu X., Xie Q., Pan X., Zhang R., Zhang X., Peng G., Zhang Y., Shen S., Tong N. (2024). Type 2 Diabetes Mellitus in Adults: Pathogenesis, Prevention and Therapy. Signal Transduct. Target. Ther..

[16] Yan S., Santoro A., Niphakis M.J., Pinto A.M., Jacobs C.L., Ahmad R., Suciu R.M., Fonslow B.R., Herbst-Graham R.A., Ngo N. (2024). Inflammation Causes Insulin Resistance in Mice via Interferon Regulatory Factor 3 (IRF3)-Mediated Reduction in FAHFA Levels. Nat. Commun..

[17] Zeng Y., Li Y., Jiang W., Hou N. (2024). Molecular Mechanisms of Metabolic Dysregulation in Diabetic Cardiomyopathy. Front. Cardiovasc. Med..

[18] Kruczkowska W., Gałęziewska J., Kciuk M., Gielecińska A., Płuciennik E., Pasieka Z., Zhao L.-Y., Yu Y.-J., Kołat D., Kałuzińska-Kołat Ż. (2024). Senescent Adipocytes and Type 2 Diabetes—Current Knowledge and Perspective Concepts. Biomol. Concepts.

[19] Dahik V.D., Kc P., Materne C., Reydellet C., Lhomme M., Cruciani-Guglielmacci C., Denom J., Bun E., Ponnaiah M., Deknuydt F. (2024). ABCG1 Orchestrates Adipose Tissue Macrophage Plasticity and Insulin Resistance in Obesity by Rewiring Saturated Fatty Acid Pools. Sci. Transl. Med..

[20] Suzuki K., Hatzikotoulas K., Southam L., Taylor H.J., Yin X., Lorenz K.M., Mandla R., Huerta-Chagoya A., Melloni G.E.M., Kanoni S. (2024). Genetic Drivers of Heterogeneity in Type 2 Diabetes Pathophysiology. Nature.

[21] Diamanti K., Cavalli M., Pereira M.J., Pan G., Castillejo-López C., Kumar C., Mundt F., Komorowski J., Deshmukh A.S., Mann M. (2022). Organ-Specific Metabolic Pathways Distinguish Prediabetes, Type 2 Diabetes, and Normal Tissues. Cell Rep. Med..

[22] Rahman M.M., Liu F.F., Eckel S.P., Sankaranarayanan I., Shafiei-Jahani P., Howard E., Baronikian L., Sattler F., Lurmann F.W., Allayee H. (2022). Near-Roadway Air Pollution, Immune Cells and Adipokines among Obese Young Adults. Environ. Health.

[23] Luo J., Wang A., Zhen W., Wang Y., Si H., Jia Z., Alkhalidy H., Cheng Z., Gilbert E., Xu B. (2018). Phytonutrient Genistein Is a Survival Factor for Pancreatic β-Cells via GPR30-Mediated Mechanism. J. Nutr. Biochem..

[24] Suksri K., Semprasert N., Limjindaporn T., Yenchitsomanus P.-T., Kooptiwoot S., Kooptiwut S. (2022). Cytoprotective Effect of Genistein against Dexamethasone-Induced Pancreatic β-Cell Apoptosis. Sci. Rep..

[25] Liu D., Zhen W., Yang Z., Carter J.D., Si H., Reynolds K.A. (2006). Genistein Acutely Stimulates Insulin Secretion in Pancreatic Beta-Cells through a cAMP-Dependent Protein Kinase Pathway. Diabetes.

[26] Andrade F.D.O., Liu F., Zhang X., Rosim M.P., Dani C., Cruz I., Wang T.T.Y., Helferich W., Li R.W., Hilakivi-Clarke L. (2021). Genistein Reduces the Risk of Local Mammary Cancer Recurrence and Ameliorates Alterations in the Gut Microbiota in the Offspring of Obese Dams. Nutrients.

[27] Goh Y.X., Jalil J., Lam K.W., Husain K., Premakumar C.M. (2022). Genistein: A Review on Its Anti-Inflammatory Properties. Front. Pharmacol..

[28] Jin S., Zheng Y., Li D., Liu X., Zhu T., Wang S., Liu Z., Liu Y. (2024). Effect of Genistein Supplementation on Microenvironment Regulation of Breast Tumors in Obese Mice. Breast Cancer Res. BCR.

[29] Zhou L., Xiao X., Zhang Q., Zheng J., Li M., Deng M. (2019). A Possible Mechanism: Genistein Improves Metabolism and Induces White Fat Browning Through Modulating Hypothalamic Expression of Ucn3, Depp, and Stc1. Front. Endocrinol..

[30] Li R.-Z., Ding X.-W., Geetha T., Al-Nakkash L., Broderick T.L., Babu J.R. (2020). Beneficial Effect of Genistein on Diabetes-Induced Brain Damage in the Ob/Ob Mouse Model. Drug Des. Devel. Ther..

[31] Duan X., Li Y., Xu F., Ding H. (2021). Study on the Neuroprotective Effects of Genistein on Alzheimer’s Disease. Brain Behav..

[32] Li Y., Zhang J.-J., Chen R.-J., Chen L., Chen S., Yang X.-F., Min J.-W. (2022). Genistein Mitigates Oxidative Stress and Inflammation by Regulating Nrf2/HO-1 and NF-κB Signaling Pathways in Hypoxic-Ischemic Brain Damage in Neonatal Mice. Ann. Transl. Med..

[33] Tsalamandris S., Antonopoulos A.S., Oikonomou E., Papamikroulis G.-A., Vogiatzi G., Papaioannou S., Deftereos S., Tousoulis D. (2019). The Role of Inflammation in Diabetes: Current Concepts and Future Perspectives. Eur. Cardiol. Rev..

[34] Lim A.K. (2014). Diabetic Nephropathy—Complications and Treatment. Int. J. Nephrol. Renov. Dis..

[35] Jha R., Lopez-Trevino S., Kankanamalage H.R., Jha J.C. (2024). Diabetes and Renal Complications: An Overview on Pathophysiology, Biomarkers and Therapeutic Interventions. Biomedicines.

[36] Thomas M.C., Brownlee M., Susztak K., Sharma K., Jandeleit-Dahm K., Zoungas S., Rossing P., Groop P.-H., Cooper M.E. (2015). Diabetic Kidney Disease. Nat. Rev. Dis. Primer.

[37] Fanti P., Asmis R., Stephenson T.J., Sawaya B.P., Franke A.A. (2006). Positive Effect of Dietary Soy in ESRD Patients with Systemic Inflammation--Correlation between Blood Levels of the Soy Isoflavones and the Acute-Phase Reactants. Nephrol. Dial. Transplant..

[38] Charles C., Yuskavage J., Carlson O., John M., Tagalicud A.S., Maggio M., Muller D.C., Egan J., Basaria S. (2009). Effects of High-Dose Isoflavones on Metabolic and Inflammatory Markers in Healthy Postmenopausal Women. Menopause.

[39] Nebbioso M., Federici M., Rusciano D., Evangelista M., Pescosolido N. (2012). Oxidative Stress in Preretinopathic Diabetes Subjects and Antioxidants. Diabetes Technol. Ther..

[40] Squadrito F., Marini H., Bitto A., Altavilla D., Polito F., Adamo E.B., D’Anna R., Arcoraci V., Burnett B.P., Minutoli L. (2013). Genistein in the Metabolic Syndrome: Results of a Randomized Clinical Trial. J. Clin. Endocrinol. Metab..

[41] Braxas H., Rafraf M., Karimi Hasanabad S., Asghari Jafarabadi M. (2019). Effectiveness of Genistein Supplementation on Metabolic Factors and Antioxidant Status in Postmenopausal Women with Type 2 Diabetes Mellitus. Can. J. Diabetes.

[42] Ruiz Esparza Cisneros J., Vasconcelos-Ulloa J.J., González-Mendoza D., Beltrán-González G., Díaz-Molina R. (2020). Effect of Dietary Intervention with a Legume-Based Food Product on Malondialdehyde Levels, HOMA Index, and Lipid Profile. Endocrinol. Diabetes Nutr..

[43] Guevara-Cruz M., Godinez-Salas E.T., Sanchez-Tapia M., Torres-Villalobos G., Pichardo-Ontiveros E., Guizar-Heredia R., Arteaga-Sanchez L., Gamba G., Mojica-Espinosa R., Schcolnik-Cabrera A. (2020). Genistein Stimulates Insulin Sensitivity through Gut Microbiota Reshaping and Skeletal Muscle AMPK Activation in Obese Subjects. BMJ Open Diabetes Res. Care.

[44] Domínguez-López I., Lozano-Castellón J., Vallverdú-Queralt A., Jáuregui O., Martínez-González M.Á., Hu F.B., Fitó M., Ros E., Estruch R., Lamuela-Raventós R.M. (2023). Urinary Metabolomics of Phenolic Compounds Reveals Biomarkers of Type-2 Diabetes within the PREDIMED Trial. Biomed. Pharmacother..

[45] Franke A.A., Custer L.J. (1994). High-Performance Liquid Chromatographic Assay of Isoflavonoids and Coumestrol from Human Urine. J. Chromatogr. B Biomed. Appl..

[46] Hutchins A.M., Slavin J.L., Lampe J.W. (1995). Urinary Isoflavonoid Phytoestrogen and Lignan Excretion after Consumption of Fermented and Unfermented Soy Products. J. Am. Diet. Assoc..

[47] Hutchins A.M., Lampe J.W., Martini M.C., Campbell D.R., Slavin J.L. (1995). Vegetables, Fruits, and Legumes: Effect on Urinary Isoflavonoid Phytoestrogen and Lignan Excretion. J. Am. Diet. Assoc..

[48] Xu X., Harris K.S., Wang H.J., Murphy P.A., Hendrich S. (1995). Bioavailability of Soybean Isoflavones Depends upon Gut Microflora in Women. J. Nutr..

[49] Barnes S., Sfakianos J., Coward L., Kirk M. (1996). Soy Isoflavonoids and Cancer Prevention. Underlying Biochemical and Pharmacological Issues.. Adv. Exp. Med. Biol..

[50] Tew B.Y., Xu X., Wang H.J., Murphy P.A., Hendrich S. (1996). A Diet High in Wheat Fiber Decreases the Bioavailability of Soybean Isoflavones in a Single Meal Fed to Women. J. Nutr..

[51] Xu X., Wang H.J., Murphy P.A., Hendrich S. (2000). Neither Background Diet nor Type of Soy Food Affects Short-Term Isoflavone Bioavailability in Women. J. Nutr..

[52] Karr S.C., Lampe J.W., Hutchins A.M., Slavin J.L. (1997). Urinary Isoflavonoid Excretion in Humans Is Dose Dependent at Low to Moderate Levels of Soy-Protein Consumption. Am. J. Clin. Nutr..

[53] Watanabe S., Yamaguchi M., Sobue T., Takahashi T., Miura T., Arai Y., Mazur W., Wähälä K., Adlercreutz H. (1998). Pharmacokinetics of Soybean Isoflavones in Plasma, Urine and Feces of Men after Ingestion of 60 g Baked Soybean Powder (Kinako). J. Nutr..

[54] Doerge D.R., Chang H.C., Churchwell M.I., Holder C.L. (2000). Analysis of Soy Isoflavone Conjugation in Vitro and in Human Blood Using Liquid Chromatography-Mass Spectrometry. Drug Metab. Dispos. Biol. Fate Chem..

[55] Vänttinen K., Moravcova J. (2001). Transdermal Absorption of Phytoestrogens. Die Pharm..

[56] Busby M.G., Jeffcoat A.R., Bloedon L.T., Koch M.A., Black T., Dix K.J., Heizer W.D., Thomas B.F., Hill J.M., Crowell J.A. (2002). Clinical Characteristics and Pharmacokinetics of Purified Soy Isoflavones: Single-Dose Administration to Healthy Men. Am. J. Clin. Nutr..

[57] Setchell K.D.R., Brown N.M., Zimmer-Nechemias L., Brashear W.T., Wolfe B.E., Kirschner A.S., Heubi J.E. (2002). Evidence for Lack of Absorption of Soy Isoflavone Glycosides in Humans, Supporting the Crucial Role of Intestinal Metabolism for Bioavailability. Am. J. Clin. Nutr..

[58] Richelle M., Pridmore-Merten S., Bodenstab S., Enslen M., Offord E.A. (2002). Hydrolysis of Isoflavone Glycosides to Aglycones by Beta-Glycosidase Does Not Alter Plasma and Urine Isoflavone Pharmacokinetics in Postmenopausal Women. J. Nutr..

[59] Zubik L., Meydani M. (2003). Bioavailability of Soybean Isoflavones from Aglycone and Glucoside Forms in American Women. Am. J. Clin. Nutr..

[60] Takimoto C.H., Glover K., Huang X., Hayes S.A., Gallot L., Quinn M., Jovanovic B.D., Shapiro A., Hernandez L., Goetz A. (2003). Phase I Pharmacokinetic and Pharmacodynamic Analysis of Unconjugated Soy Isoflavones Administered to Individuals with Cancer. Cancer Epidemiol. Biomark. Prev..

[61] Faughnan M.S., Hawdon A., Ah-Singh E., Brown J., Millward D.J., Cassidy A. (2004). Urinary Isoflavone Kinetics: The Effect of Age, Gender, Food Matrix and Chemical Composition. Br. J. Nutr..

[62] Maubach J., Depypere H.T., Goeman J., Van der Eycken J., Heyerick A., Bracke M.E., Blondeel P., De Keukeleire D. (2004). Distribution of Soy-Derived Phytoestrogens in Human Breast Tissue and Biological Fluids. Obstet. Gynecol..

[63] Bolca S., Urpi-Sarda M., Blondeel P., Roche N., Vanhaecke L., Possemiers S., Al-Maharik N., Botting N., De Keukeleire D., Bracke M. (2010). Disposition of Soy Isoflavones in Normal Human Breast Tissue. Am. J. Clin. Nutr..

[64] Fischer L., Mahoney C., Jeffcoat A.R., Koch M.A., Thomas B.E., Valentine J.L., Stinchcombe T., Boan J., Crowell J.A., Zeisel S.H. (2004). Clinical Characteristics and Pharmacokinetics of Purified Soy Isoflavones: Multiple-Dose Administration to Men with Prostate Neoplasia. Nutr. Cancer.

[65] Rannikko A., Petas A., Rannikko S., Adlercreutz H. (2006). Plasma and Prostate Phytoestrogen Concentrations in Prostate Cancer Patients after Oral Phytoestogen Supplementation. Prostate.

[66] Guy L., Védrine N., Urpi-Sarda M., Gil-Izquierdo A., Al-Maharik N., Boiteux J.-P., Scalbert A., Remesy C., Botting N.P., Manach C. (2008). Orally Administered Isoflavones Are Present as Glucuronides in the Human Prostate. Nutr. Cancer.

[67] Anupongsanugool E., Teekachunhatean S., Rojanasthien N., Pongsatha S., Sangdee C. (2005). Pharmacokinetics of Isoflavones, Daidzein and Genistein, after Ingestion of Soy Beverage Compared with Soy Extract Capsules in Postmenopausal Thai Women. BMC Clin. Pharmacol..

[68] Gardner C.D., Chatterjee L.M., Franke A.A. (2009). Effects of Isoflavone Supplements vs. Soy Foods on Blood Concentrations of Genistein and Daidzein in Adults. J. Nutr. Biochem..

[69] Vergne S., Sauvant P., Lamothe V., Chantre P., Asselineau J., Perez P., Durand M., Moore N., Bennetau-Pelissero C. (2009). Influence of Ethnic Origin (Asian v. Caucasian) and Background Diet on the Bioavailability of Dietary Isoflavones. Br. J. Nutr..

[70] Ullmann U., Metzner J., Frank T., Cohn W., Riegger C. (2005). Safety, Tolerability, and Pharmacokinetics of Single Ascending Doses of Synthetic Genistein (Bonistein) in Healthy Volunteers. Adv. Ther..

[71] Ullmann U., Oberwittle H., Grossmann M., Riegger C. (2005). Repeated Oral Once Daily Intake of Increasing Doses of the Novel Synthetic Genistein Product Bonistein in Healthy Volunteers. Planta Med..

[72] Metzner J.E., Frank T., Kunz I., Burger D., Riegger C. (2009). Study on the Pharmacokinetics of Synthetic Genistein after Multiple Oral Intake in Post-Menopausal Women. Arzneimittelforschung.

[73] Chen J., Lin H., Hu M. (2005). Absorption and Metabolism of Genistein and Its Five Isoflavone Analogs in the Human Intestinal Caco-2 Model. Cancer Chemother. Pharmacol..

[74] Langa S., Peirotén Á., Curiel J.A., de la Bastida A.R., Landete J.M. (2023). Isoflavone Metabolism by Lactic Acid Bacteria and Its Application in the Development of Fermented Soy Food with Beneficial Effects on Human Health. Foods.

[75] Coldham N.G., Darby C., Hows M., King L.J., Zhang A.Q., Sauer M.J. (2002). Comparative Metabolism of Genistin by Human and Rat Gut Microflora: Detection and Identification of the End-Products of Metabolism. Xenobiotica.

[76] Tsangalis D., Wilcox G., Shah N.P., Stojanovska L. (2005). Bioavailability of Isoflavone Phytoestrogens in Postmenopausal Women Consuming Soya Milk Fermented with Probiotic Bifidobacteria. Br. J. Nutr..

[77] McMullen M.H., Hamilton-Reeves J.M., Bonorden M.J.L., Wangen K.E., Phipps W.R., Feirtag J.M., Kurzer M.S. (2006). Consumption of Lactobacillus Acidophilus and Bifidobacterium Longum Does Not Alter Phytoestrogen Metabolism and Plasma Hormones in Men: A Pilot Study. J. Altern. Complement. Med..

[78] Larkin T.A., Price W.E., Astheimer L.B. (2007). Increased Probiotic Yogurt or Resistant Starch Intake Does Not Affect Isoflavone Bioavailability in Subjects Consuming a High Soy Diet. Nutrition.

[79] Benvenuti C., Setnikar I. (2011). Effect of *Lactobacillus sporogenes* on Oral Isoflavones Bioavailability: Single Dose Pharmacokinetic Study in Menopausal Women. Arzneimittelforschung.

[80] Yuan B., Zhen H., Jin Y., Xu L., Jiang X., Sun S., Li C., Xu H. (2012). Absorption and Plasma Disposition of Genistin Differ from Those of Genistein in Healthy Women. J. Agric. Food Chem..

[81] Smit S., Szymańska E., Kunz I., Gomez Roldan V., van Tilborg M.W.E.M., Weber P., Prudence K., van der Kloet F.M., van Duynhoven J.P.M., Smilde A.K. (2014). Nutrikinetic Modeling Reveals Order of Genistein Phase II Metabolites Appearance in Human Plasma. Mol. Nutr. Food Res..

[82] Chen Y., Xiao C.-Q., He Y.-J., Chen B.-L., Wang G., Zhou G., Zhang W., Tan Z.-R., Cao S., Wang L.-P. (2011). Genistein Alters Caffeine Exposure in Healthy Female Volunteers. Eur. J. Clin. Pharmacol..

[83] Xiao C.-Q., Chen R., Lin J., Wang G., Chen Y., Tan Z.-R., Zhou H.-H. (2012). Effect of Genistein on the Activities of Cytochrome P450 3A and P-Glycoprotein in Chinese Healthy Participants. Xenobiotica Fate Foreign Compd. Biol. Syst..

[84] Bloedon L.T., Jeffcoat A.R., Lopaczynski W., Schell M.J., Black T.M., Dix K.J., Thomas B.F., Albright C., Busby M.G., Crowell J.A. (2002). Safety and Pharmacokinetics of Purified Soy Isoflavones: Single-Dose Administration to Postmenopausal Women. Am. J. Clin. Nutr..

[85] Michael McClain R., Wolz E., Davidovich A., Bausch J. (2006). Genetic Toxicity Studies with Genistein. Food Chem. Toxicol..

[86] Miltyk W., Craciunescu C.N., Fischer L., Jeffcoat R.A., Koch M.A., Lopaczynski W., Mahoney C., Jeffcoat R.A., Crowell J., Paglieri J. (2003). Lack of Significant Genotoxicity of Purified Soy Isoflavones (Genistein, Daidzein, and Glycitein) in 20 Patients with Prostate Cancer. Am. J. Clin. Nutr..

[87] Pop E.A., Fischer L.M., Coan A.D., Gitzinger M., Nakamura J., Zeisel S.H. (2008). Effects of a High Daily Dose of Soy Isoflavones on DNA Damage, Apoptosis, and Estrogenic Outcomes in Healthy Postmenopausal Women: A Phase I Clinical Trial. Menopause.

[88] Illahi A.F., Muhammad F., Akhtar B. (2019). Nanoformulations of Nutraceuticals for Cancer Treatment. Crit. Rev. Eukaryot. Gene Expr..

[89] Pandey P., Ramniwas S., Pandey S., Verma M., Kumar R., Lakhanpal S., Khan F., Shah M.A. (2024). An Updated Review Summarizing the Pharmaceutical Efficacy of Genistein and Its Nanoformulations in Ovarian Carcinoma. Curr. Pharm. Des..

[90] Pham J., Grundmann O., Elbayoumi T. (2015). Mitochondriotropic Nanoemulsified Genistein-Loaded Vehicles for Cancer Therapy. Mitochondrial Medicine: Volume II, Manipulating Mitochondrial Function.

[91] Botet-Carreras A., Tamames-Tabar C., Salles F., Rojas S., Imbuluzqueta E., Lana H., Blanco-Prieto M.J., Horcajada P. (2021). Improving the Genistein Oral Bioavailability via Its Formulation into the Metal-Organic Framework MIL-100(Fe). J. Mater. Chem. B.

[92] Iqbal F.M., Rodríguez-Nogales C., Boulens N., Delie F. (2024). Formulation and Optimization of Transferrin-Modified Genistein Nanocrystals: In Vitro Anti-Cancer Assessment and Pharmacokinetic Evaluation. Int. J. Pharm..

[93] Phan V., Walters J., Brownlow B., Elbayoumi T. (2013). Enhanced Cytotoxicity of Optimized Liposomal Genistein via Specific Induction of Apoptosis in Breast, Ovarian and Prostate Carcinomas. J. Drug Target..

[94] Yamamoto S., Ohta A., Hossain F., Anjani G., Asakawa H., Asakawa T. (2019). Solubilization of Genistein in Phospholipid Vesicles and Their Atioxidant Capacity. J. Oleo Sci..

[95] Komeil I.A., Abdallah O.Y., El-Refaie W.M. (2022). Surface Modified Genistein Phytosome for Breast Cancer Treatment: In-Vitro Appraisal, Pharmacokinetics, and In-Vivo Antitumor Efficacy. Eur. J. Pharm. Sci..

[96] Yan H., Li Y., Li S., Wu D., Xu Y., Hu J. (2023). Phosphatidylserine-Functionalized Liposomes-in-Microgels for Delivering Genistein to Effectively Treat Ulcerative Colitis. J. Mater. Chem. B.

[97] Sharma S., Gupta P., Kawish S.M., Ahmad S., Iqbal Z., Vohora D., Kohli K. (2024). Novel Chitosan-Coated Liposomes Coloaded with Exemestane and Genistein for an Effective Breast Cancer Therapy. ACS Omega.

[98] Gao R., Lin P., Yang W., Fang Z., Gao C., Cheng B., Fang J., Yu W. (2024). Bio-Inspired Nanodelivery Platform: Platelet Membrane-Cloaked Genistein Nanosystem for Targeted Lung Cancer Therapy. Int. J. Nanomed..

[99] Lopes de Azambuja C.R., dos Santos L.G., Rodrigues M.R., Rodrigues R.F.M., da Silveira E.F., Azambuja J.H., Flores A.F.C., Horn A.P., Dora C.L., Muccillo-Baisch A.L. (2015). Physico-Chemical Characterization of Asolectin-Genistein Liposomal System: An Approach to Analyze Its in Vitro Antioxidant Potential and Effect in Glioma Cells Viability. Chem. Phys. Lipids.

